# Integrating Network Pharmacology and Experimental Validation to Explore the Key Mechanism of Gubitong Recipe in the Treatment of Osteoarthritis

**DOI:** 10.1155/2022/7858925

**Published:** 2022-06-08

**Authors:** Guang-yao Chen, Xiao-yu Liu, Jing Luo, Xin-bo Yu, Yi Liu, Qing-wen Tao

**Affiliations:** ^1^Beijing University of Chinese Medicine, Beijing 100029, China; ^2^Department of TCM Rheumatology, China-Japan Friendship Hospital, Beijing 100029, China; ^3^Beijing Key Lab for Immune-Mediated Inflammatory Diseases, China-Japan Friendship Hospital, Beijing 100029, China

## Abstract

**Background:**

Gubitong Recipe (GBT) is a prescription based on the Traditional Chinese Medicine (TCM) theory of tonifying the kidney yang and strengthening the bone. A previous multicentral randomized clinical trial has shown that GBT can effectively relieve joint pain and improve quality of life with a high safety in treating osteoarthritis (OA). This study is aimed at elucidating the active compounds, potential targets, and mechanisms of GBT for treating OA.

**Method:**

The network pharmacology method was used to predict the key active compounds, targets, and mechanisms of GBT in treating OA. An OA rat model was established with Hulth surgery, and the pathological changes of articular cartilage were observed to evaluate the effects of GBT. Chondrocytes were stimulated with LPS to establish in vitro models, and key targets and mechanisms predicted by network pharmacology were verified via qRT-PCR, ELISA, western blot, and immunofluorescence. The Contribution Index Model and molecular docking were used to determine the key active compounds of GBT and the major nodes affecting predicted pathways.

**Result:**

A total of 500 compounds were acquired from related databases, where 87 active compounds and their 254 corresponding targets were identified. 2979 OA-related genes were collected from three databases, 150 of which were GBT-regulating OA genes. The compound-target network weight analysis and PPI results showed that IL-6 and PGE_2_ are key targets of GBT in treating OA. KEGG results showed that PI3K/AKT, Toll-like receptor, NF*κ*B, TNF, and HIF-1 are the key signaling pathways. An in vivo experiment showed that GBT could effectively suppress cartilage degradation of OA rats. In vitro experiments demonstrated that GBT can inhibit the key targets of KEGG-related pathways. Molecular-docking results suggested that luteolin, licochalcone A, and *β*-carotene were key targets of GBT, and the mechanisms may be associated with the NF*κ*B signaling pathway. Blockage experiments showed that the NF*κ*B pathway is the key pathway of GBT in treating OA.

**Conclusion:**

This study verified that GBT can effectively protect articular cartilage through multitarget and multipathway, and its inhibitory effect on the NF*κ*B pathway is the most key mechanism in treating OA.

## 1. Introduction

Osteoarthritis (OA) is a degenerative disease characterized by degeneration of the cartilage, which is closely related to age, joint trauma, obesity, and genetic factors [1, 2]. Previous studies showed that OA affects more than one-third of the elderly, and its incidence keeps escalating due to the global aging population and obesity epidemic [3, 4]. The imbalance between the synthesis and hydrolysis of the cartilage matrix, which leads to structural changes of the cartilage, is a crucial cause of OA [5]. Senescence and inflammatory response of chondrocytes are key to the imbalance of the cartilage matrix. Thus, the intervention in chondrocytes was considered essential to the treatment of OA.

According to Traditional Chinese Medicine (TCM) theory, OA is considered to be a bone arthralgia syndrome (Gubi) disease attributed to deficiency of kidney yang. Clinical investigation shows that most OA patients, especially elderly patients, have kidney-yang deficiency syndromes. Gubitong Recipe (GBT) is a prescription formulated based on the TCM theory of tonifying the kidney yang and strengthening the bone. Our previous randomized clinical trials (RCT) have shown that GBT can more effectively decrease the Western Ontario and McMaster Universities Osteoarthritis Index (WOMAC) score and clinical symptoms of the visual analogue scale (VAS) of OA patients in comparison with glucosamine hydrochloride, reduce the amount of diclofenac sodium use, and has no obvious side effects [6].

Network pharmacology is a new research method, which elucidates the potential mechanism of specific disease through database analysis and target prediction [7, 8]. Molecular docking is an in silico approach to identify the most effective compounds and proteins from a good deal of compounds and proteins [9, 10]. This study is designed to further reveal the mechanism of GBT against OA; the network pharmacology method was used to predict key targets and pathways of GBT. Molecular docking was performed to validate the interaction between key compounds and core proteins of key pathways. In vivo and in vitro experiments were conducted to verify the predicted results of network pharmacology.

## 2. Materials and Methods

### 2.1. Collection of Active Compounds of GBT

All compounds of the eight botanical drugs of GBT (*Drynaria fortunei* (Kunze) J.Sm. (Gusuibu, 20 g), *Epimedium brevicornum* Maxim. (Yinyanghuo, 15 g), *Psoralea corylifolia* L. (Buguzhi, 15 g), *Eucommia ulmoides* Oliv. (Duzhong, 30 g), *Cibotium barometz* (L.) J.Sm. (Gouji, 30 g), *Bolbostemma paniculatum* (Maxim.) Franquet. (Tubeimu, 20 g), *Sinomenium acutum* (Thunb.) Rehd. et Wils. (Qingfengteng, 30 g), *Spatholobus suberectus* Dunn. (Jixueteng, 30 g)) were identified from the following four databases: Traditional Chinese Medicines for Systems Pharmacology Database and Analysis Platform (TCMSP) [11], Yet another Traditional Chinese Medicine database (YaTCM) [12], Traditional Chinese Medicine Database@Taiwan [13], and Traditional Chinese Medicine Integrated Database (TCMID) [14]. Results from each database were combined and duplicates were removed. The properties of GBT-related compounds, including molecular weight, oral bioavailability, and drug-likeness, were collected from the TCMSP database. The chemical structural formulas were identified from the PubChem database [15].

Oral bioavailability and drug-likeness scores were set as the parameters for compound screening. Oral bioavailability represents the degree that a compound actually enters human systemic circulation. Drug-likeness indicates the similarity between a compound and the known drugs, and a compound with a high drug-likeness score indicates chemical suitability for drug development. In this study, oral bioavailability ≥ 30% and drug − likeness ≥ 0.18 were the criteria for the active compound screening [16].

### 2.2. Collection of Active Compounds of GBT and OA Targets

Corresponding targets of the active compounds of GBT were obtained by two sources from the TCMSP database (http://lsp.nwu.edu.cn/tcmsp.php). First, the HIT database (https://ngdc.cncb.ac.cn/databasecommons) was used to screen out the experimental validated targets corresponding to the above compounds [17]. If the compound was not available in the HIT database, the SysDT model was used to predict the corresponding targets [18]. The PDBIDs (Protein Data Bank Identity Documents) of the targets were input into the UniProt database to normalize the target name. The GeneCards (https://www.genecards.org/) [19], DisGeNET (https://www.disgenet.org/) [20], and OMIM (https://www.omim.org/) [21] databases were used to collect OA-related targets, which were identified with “osteoarthritis” as the keyword.

### 2.3. PPI Network Construction

The common targets between GBT and OA were analyzed by a protein-protein interaction (PPI) network using the STRING database (https://string-db.org/,version 11.0) [22]. Interaction scores greater than 0.4 were considered statistically significant. The results were visualized with Cytoscape 3.7.2, and a bar graph was generated to show the number of edges linked with the node.

### 2.4. Compound-Target Network Construction

The compound-target network involves GBT active compounds, intersection genes of active compounds, and OA potential targets of GBT active compounds. The compound-target network was constructed and visualized with Cytoscape 3.7.2 on the basis of the above data [23]. Pink nodes denote intersection genes of GBT and OA while blue nodes denote GBT active compounds. The NetworkAnalyzer Cytoscape module was used to analyze the network topological properties [24].

### 2.5. Development of the Contribution Index Model

The Contribution Index Model was established to get the effective compounds, which could be used to illustrate the key active compounds and targets of GBT in the therapy of OA. Given *n* and cumulative contribution rates, the number of targets enriched is *W*_*i*_, the coverage rate of the targets in the compound-target network compared to the pathogenic gene is *P*_*i*_, and the total restriction condition *C* is the number of the compound-target network which was used to calculate the Contribution Index. Select the appropriate compound-target network to calculate the contribution rate based on the coverage of targets in the compound-target network compared to that in pathogenic genes. When the Contribution Index reaches or exceeds 90%, the iterative cumulative calculation of this model will be terminated. There are only two options for each *i*: included or not; that is, *i* can only be included in the cumulative probability once. The model of this problem can be expressed as the following 0/1 integer programming model:

Subject to:

(1) $$\begin{array}{c} \max\;f\left(x_{1},x_{2},\cdots ,x_{n}\right)=\overset{n}{\underset{i=1}{\sum }}c_{i}x_{i},\\ \text{s}.\text{t}.\left\{\begin{array}{c} \overset{n}{\underset{i=1}{\sum }}w_{i}x_{i}\leq p_{i},\\ x_{i}\left\{0,1\right\}\left(i=1,2\cdots n\right), \end{array}\right. \end{array}$$

where *x*_*i*_ is the decision variable of 0-1, *x*_*i*_ = 1 represents that the item is chosen into the knapsack, and *x*_*i*_ = 0 represents that the item is not selected into the knapsack.

### 2.6. Gene Ontology and Pathway Enrichment Analysis

Gene Ontology (GO) analysis was used to classify the GO functional annotations [25]. The Database for Annotation, Visualization, and Integrated Discovery (DAVID, https://david.ncifcrf.gov/,version 6.8) was used for GO enrichment analysis. Biological processes were screened out, and a bar chart was generated. Pathway enrichment analysis was performed to identify highly enriched pathways associated with OA. Significant pathways that were potentially regulated by active compounds were evaluated and calculated with the Kyoto Encyclopedia of Genes and Genomes (KEGG) pathway enrichment analysis [26]. Pathways satisfying *p* < 0.05 were considered statistically significant.

### 2.7. Molecular Docking of Active Compound and Key Target

Molecular docking was used to unravel the binding affinities between proteins and ligands [27]. Key compounds of GBT and core proteins of key pathways were selected for molecular docking. In this study, structures of the ligands were downloaded from the RCSB PDB database (https://www1.rcsb.org/) and saved in PDB format. Structures of the key compounds of GBT were downloaded from the PubChem database (https://pubchem.ncbi.nlm.nih.gov/) and were converted into MOL2 format using Open Babel GUI. AutoDockTools (version 1.5.6) was used to conduct molecular docking between the key compounds of GBT and the core proteins, including identifying binding sites and calculating binding energy [28]. AutoDock exported all information as pdbqt files. The binding sites of the maximum binding energy were chosen for comparison and visualization. These pdbqt files and pdb files of corresponding proteins were inputted into PyMOL to draw figures in png format. The binding affinities of key compounds and core proteins were calculated and displayed as a heat map with assistance of the R language “heat map” function.

### 2.8. Experimental Verification

#### 2.8.1. Reagents

0.25% trypsin-EDTA, DMEM medium, phosphate-buffered saline (PBS), fetal bovine serum (FBS), and collagenase II were purchased from Gibco. Lipopolysaccharides (LPS), penicillin-streptomycin, poly-L-lysine, and Triton X-100 were purchased from Sigma. Electrochemiluminescence (ECL) luminous fluid and polyvinylidene difluoride (PVDF) membranes were purchased from Millipore. Animal-free Blocking Solution was purchased from Cell Signaling. RIPA lysis buffer, phenylmethanesulfonyl fluoride (PMSF), Tween-20, EDTA-Na_2_, Tris, glycine, sodium dodecyl sulfate (SDS), and protein phosphatase inhibitor were purchased from Solarbio Life Science. SYBR Green real-time PCR master mix was purchased from Toyobo. Fluorescent mounting medium with DAPI (4,6-diamidino-2-phenylindole) was purchased from Zhongshan Jinqiao Biotechnology. Polymerase chain reaction (PCR) primers for rat IL-6, TNF-*α*, HIF-*α*, and *β*-actin were synthesized by TSINGKE Biotechnology Co., Ltd. (primer sequences are listed in Table 1).

Rat IL-6 enzyme-linked immunosorbent assay (ELISA) kits (CRE0005), rat IL-1*β* ELISA kits (CRE0006), and rat TNF-*α* ELISA kits (CRE0003) were purchased from Beijing 4A Biotech. Rat IL-18 ELISA kit (ERC010.48) was purchased from NEOBIOSCIENCE. Prostaglandin E_2_ (PGE_2_) ELISA kit (EIA-5811) was purchased from DRG International. CellTiter 96® AQueous One Solution Cell Proliferation Assay (G3582) and Reverse Transcription System (A3500) were purchased from Promega. HiPure Total RNA Mini Kit (R4111-02) was purchased from Magen. PAGE Gel Fast Preparation Kit (PG112) was purchased from Shanghai Epizyme Biotechnology. Nuclear Extraction Kit, Hematoxylin-Eosin/HE Staining Kit, and Modified Safranine O-Fast Green FCF Cartilage Stain Kit were purchased from Solarbio Life Science. Coimmunoprecipitation kit was purchased from Absin.

#### 2.8.2. Preparation of GBT

Gusuibu 20 g, Yinyanghuo 15 g, Buguzhi 15 g, Duzhong 30 g, Gouji 30 g, Tubeimu 20 g, Qingfengteng 30 g, and Jixueteng 30 g were purchased from the TCM pharmacy of the China-Japan Friendship Hospital. The botanical drugs were added to 1.9 L water and decocted for 1 h and filtered. The above steps were repeated three times, and the filtrate was mixed together before being evaporated under reduced pressure to 500 mL. The concentrate was placed at 4°C for 12 h, the filtrate was centrifuged, and the supernatant was collected. The resulting supernatant was boiled, and anhydrous alcohol was added until an alcohol content of 70% was achieved. After cooling, the mixture was centrifuged, and the supernatant was collected, concentrated under reduced pressure, freeze-dried, and weighed.

#### 2.8.3. Determination of GBT Representative Compounds

The representative compounds of each botanical drug of GBT were collected from the Chinese Pharmacopoeia (2020 Edition) and related literature for quality control. 20 mg of GBT sample was added to 1 mL of deionized water for ultrasound for 30 min and centrifuged at 12000 r/min for 10 min to obtain the supernatant. The supernatant was filtrated by a 0.22 *μ*m membrane for sample detection. The key compounds of GBT were detected by liquid chromatography coupled with mass spectrometry (LC-MS). The results were compared with the MassBank online spectral database (https://massbank.eu/MassBank/), the ReSpect DB (http://spectra.psc.riken.jp), and the GNPS platform (https://gnps.ucsd.edu/).

#### 2.8.4. In Vivo Experiment Verification

Twenty-four 6-week-old SPF SD male rats were purchased from the Beijing Sipeifu Biotechnology Co., Ltd. for in vivo experiment intervention. All rats were housed in an animal facility according to the National Standards for Laboratory Animals of China (GB 14925-2010). The experiment was approved by the Animal Ethic Committee of the China-Japan Friendship Hospital (no. zryhyy-21-21-05-02).

After adaptive feeding for seven days, the rats were divided into the normal group, OA model group, GBT intervention group, and glucosamine hydrochloride intervention group using the random number table method. The OA model group, the GBT intervention group, and the glucosamine hydrochloride intervention group were used to establish the OA rat model with the Hulth method. The specific methods are as follows. Rats were anaesthetized initially with isoflurane. The right knee surface of the rats was processed for skin preparation and sterilized with alcohol. The skin and patellar ligament of rats were cut with a scalpel, the medial meniscus was removed, and the medial collateral ligament and the anterior and posterior cruciate ligaments were further cut off. After confirming with the positive drawer test of the knee joint of rats, the skin and patellar ligament were sutured layer by layer. After the operation, each rat was injected with 200,000 units of penicillin for 3 days to avoid infection.

Three days after the Hulth surgery, the GBT solution was equivalent to 1.44 g/mL of the raw botanical drugs, and glucosamine hydrochloride was configured to 13.8 mg/mL solution. The normal group and OA model group were given 1 mL saline for every 100 g body weight, and the GBT intervention group and glucosamine hydrochloride intervention group were given 1 mL corresponding drug for every 100 g body weight. The rats were given gavage at 8:00 every morning for 28 days. After the experiment, the rats were sacrificed in a carbon dioxide chamber. The right knee joints were prepared into pathology slices, stained with hematoxylin and eosin and safranin O/fast green, and the samples were pathologically evaluated.

#### 2.8.5. In Vitro Experiment Verification

Twelve 5-day-old suckling rats (purchased from the Beijing SIBF Biotechnology Co., Ltd.,) were used for chondrocyte isolation. The rats were sacrificed with carbon dioxide and then sterilized with 75% alcohol for 10 min. The knee cartilage of rats was separated with a scalpel and dissociated enzymatically with 0.25% EDTA-trypsin for 1 hour to remove the connective tissue on the surface. After dissociation, 10% FBS solution in PBS was used to neutralize trypsin dissociation and washed with PBS for 3 times. The cartilage tissue was then dissociated enzymatically with 0.2% type II collagenase prepared in DMEM medium for 4 h, and the suspension containing cells was obtained by filtration with a cell screen. The suspension was centrifuged (4°C, 1700 r/min, 5 min), and the supernatant was discarded to obtain chondrocytes. The obtained chondrocytes were added to DMEM complete medium (89% DMEM basic medium, 10% FBS, and 1% penicillin-streptomycin) and cultured in a 10 cm culture dish coated with polylysine at 37°C in a 5% carbon dioxide cell incubator. When overgrown, the cells were digested with 0.25% EDTA-trypsin, and the passage was carried out at a ratio of 1 : 2. During in vitro culture, chondrocytes will gradually lose their phenotypes due to dedifferentiation, so primary chondrocytes were cultured for no more than 3 passages for experiment use.

#### 2.8.6. Preparation of Pathological Sections and Pathological Evaluation

Knee joints were separated with a bone forceps, cutting 1 cm above and below the knee joint. We remove as much muscle tissue as possible without damaging the articular and then fix it with 4% paraformaldehyde for 72 h. After 8 weeks of decalcification with a 10% EDTA solution, the joint was cut along the mid-sagittal plane, rinsed with distilled water and placed in an embedded box. Dehydration, clearing, infiltration, and slicing were done sequentially. Sections were stained with hematoxylin and eosin for evaluation of the basic morphology of cartilage, and the matrix proteoglycan of the cartilage was observed with safranin O/fast green staining. Structures of the chondrocytes were detected with light microscopy and evaluated using Mankin's score (Table 2) [29].

#### 2.8.7. Identification of Chondrocytes

As a specific marker of mature chondrocytes, type II collagen is often selected as a biomarker for chondrocytes [30]. Chondrosarcomas are regarded as malignant tumor cells that originate from chondrocytes. Leibovitz isolated chondrosarcoma cells from a primary grade II chondrosarcoma of the right humerus from a 72-year-old woman in 1982 and successfully established the SW1353 cell line [31]. SW1353 cells and human chondrocytes are similar in phenotypes, and thus, SW1353 cells are often applied to in vitro experiments on OA [32, 33]. However, SW1353 has no characteristics of secreting type II collagen and was therefore used as control cells for the identification of chondrocytes.

The isolated chondrocytes were first subjected to immunofluorescent staining using a type II collagen antibody to determine whether or not the chondrocytes possessed the characteristic of expressing and secreting type II collagen [34]. Further, the isolated chondrocytes were detected by flow cytometry assay to define the proportion of chondrocytes positive for type II collagen, while chondrocytes without the addition of the type II collagen antibody and SW1353 cells were used as the negative control.

#### 2.8.8. MTS Assay

Cell viability was detected with the MTS assay. Chondrocytes were diluted to a concentration of 1 × 10^5^ cells/mL and then planted at 100 *μ*L per well on a 96-well plate. 0 *μ*g/mL, 50 *μ*g/mL, 100 *μ*g/mL, 200 *μ*g/mL, and 250 *μ*g/mL GBT solutions were added to each well, and cells were cultured for 12 h. After discarding the medium, the cells were washed twice with PBS. 100 *μ*L of DMEM medium was added to each well, and cells were cultured for 2 h before 20 *μ*L MTS was added. The chondrocytes were then incubated for another 30 min. A microplate reader (Molecular Devices, USA) was used to measure the optical density (OD) of each well at 490 nm. The following formula was used to calculate cell viability: viability (%) = 100 × (OD of treated sample − OD of medium)/(OD of control sample − OD of medium).

#### 2.8.9. RNA Isolation and qRT-PCR

A HiPure Total RNA Mini Kit was used to extract and purify total RNA using an adsorption column and collection pipes. A NanoDrop spectrophotometer (Thermo Scientific, USA) was used to detect RNA concentration and quality. 1 *μ*g total RNA was used to construct a transcription system (A3500, Promega, USA) and was then transcribed into cDNA according to the manufacturer's instruction.

20 *μ*L of the reaction system included SYBR Green real-time PCR master mix (10 *μ*L), cDNA (2 *μ*L), gene forward and reverse primer (10 *μ*mol/L, 0.8 *μ*L), and distilled water (7.2 *μ*L). Real-time PCR was performed on a QuantStudio Real-Time PCR cycler with the following protocol: preheated at 95°C for 60 s, then heated at 95°C for 15 s, 60°C for 15 s, and 72°C for 45 s as a cycle; a total of 40 cycles were completed, and finally, the CT value was obtained. The relative expression was analyzed based on the 2^-Ct^ method.

#### 2.8.10. Western Blotting

Before the experiment, 10% SDS-PAGE gel was configured with a PAGE Gel Fast Preparation Kit based on the kit's instructions. Specific information and dilution rates of all antibodies are presented in Table 3.

First, cells were rinsed twice with PBS. Each well was added with 250 *μ*L RIPA lysate (containing 1% PMSF and 1% phosphatase inhibitor), gently blown for 5-10 times, and lysed on ice for 25 min. The lysate was centrifuged (4°C, 10000 r/min, 5 min), and supernatants were collected. A bicinchoninic acid (BCA) assay kit was used to detect total protein concentration. According to the concentration test results, samples were adjusted to the same protein concentration with the RIPA lysate. A 5x loading buffer (dilution 4 : 1) was added, then boiled for 5 min, and then centrifuged (4°C, 10000 r/min, 5 min).

40 *μ*g of proteins was added into each well of the SDS-PAGE gel and labeled with a marker protein. Electrophoresis was performed at 150 V until the bromophenol blue reached the bottom of the gel. The PVDF membrane was activated with methanol for 40 s before transfer. A Mini Gel Holder Cassette, filtrate paper, PVDF membrane, and SDS-PAGE gel were prepared to make a sandwich transfer system and electrophoresis for 55 min at 70 V. Proteins in the gel were transferred to PVDF membranes and then incubated with a blocking solution for 1 h at room temperature. The PVDF membranes were then washed with TBST, added with diluted primary antibodies, and incubated at 4°C overnight. After that, the PVDF membranes were washed with TBST for five times, added with diluted secondary antibodies, and incubated for 1.5 h at room temperature. All incubations were performed on an oscillating shaker. Finally, the PVDF membranes were washed for five times with TBST, and the samples were detected with a chemiluminescence system using ECL luminous fluid.

#### 2.8.11. ELISA

Cell culture media were collected after completion of cell intervention and then centrifugated (4°C, 3000 r/min, 5 min), and supernatants were obtained for ELISA detection. The concentrations of IL-6, IL-1*β*, IL-18, TNF-*α*, and PGE_2_ in the supernatant were determined using a commercial ELISA kit according to the manufacturers' instructions (IL-6, IL-1*β*, IL-18, and TNF-*α* were monitored using the double antibody sandwich method, and PGE_2_ was monitored using the competitive method). The OD value of each standard and sample pore was detected at 450 nm wavelength with a microplate reader, with a reference wavelength of 650 nm. A model of standard curve was constructed using CurveExpert 1.4 software, and the concentrations of the standard pore sample were calculated using the OD value.

#### 2.8.12. Immunofluorescence

After being washed with PBS, the cells were fixed with 4% paraformaldehyde. PBS/0.1% Triton X-100 was used to penetrate the cytomembrane. After being blocked with an animal-free blocking solution for 1 h at room temperature, the primary antibody (type II collagen of dilution at 1 : 100 and NF*κ*B p65 of dilution at 1 : 200) was added and incubated at 4°C overnight. After discarding the primary antibody, Alexa Fluor 488-conjugated goat antirabbit IgG (H+L) (1 : 100 dilution) was added and incubated at room temperature for 1 h; then, the plate was covered with a fluorescent mounting medium with DAPI. An inverted fluorescence microscope (Zeiss, Germany) was used to observe the location of the antibody and DAPI to determine the location of the target protein and nucleus.

#### 2.8.13. Flow Cytometry Assay

Chondrocytes and SW1353 cells were subjected to digestion of *EDTA*-free trypsin, followed by the neutralization of the DEME complete medium. Type II collagen antibodies (1 : 100 dilution) were injected into dishes for 1 h incubation after the cells were fixed and the membrane was broken. Finally, these cells were washed with 1x buffer, followed by the addition of Alexa Fluor 488-conjugated goat antirabbit IgG (H+L) (1 : 100 dilution) to cells (1 : 50 dilution). After 30 min incubation under a light-free circumstance, these prepared cells were assayed with a flow cytometer (BD FACSCanto II, USA).

### 2.9. Statistical Analysis

Continuous variables are presented as the mean values ± standard deviation (SD). Student's *t* test was used to assess the differences between the two groups. ANOVA and Dunnett's test were used for comparisons of multiple groups (GraphPad Prism 4). A *p* value < 0.05 was considered to indicate a statistically significant difference.

## 3. Results

**Figure 1:**
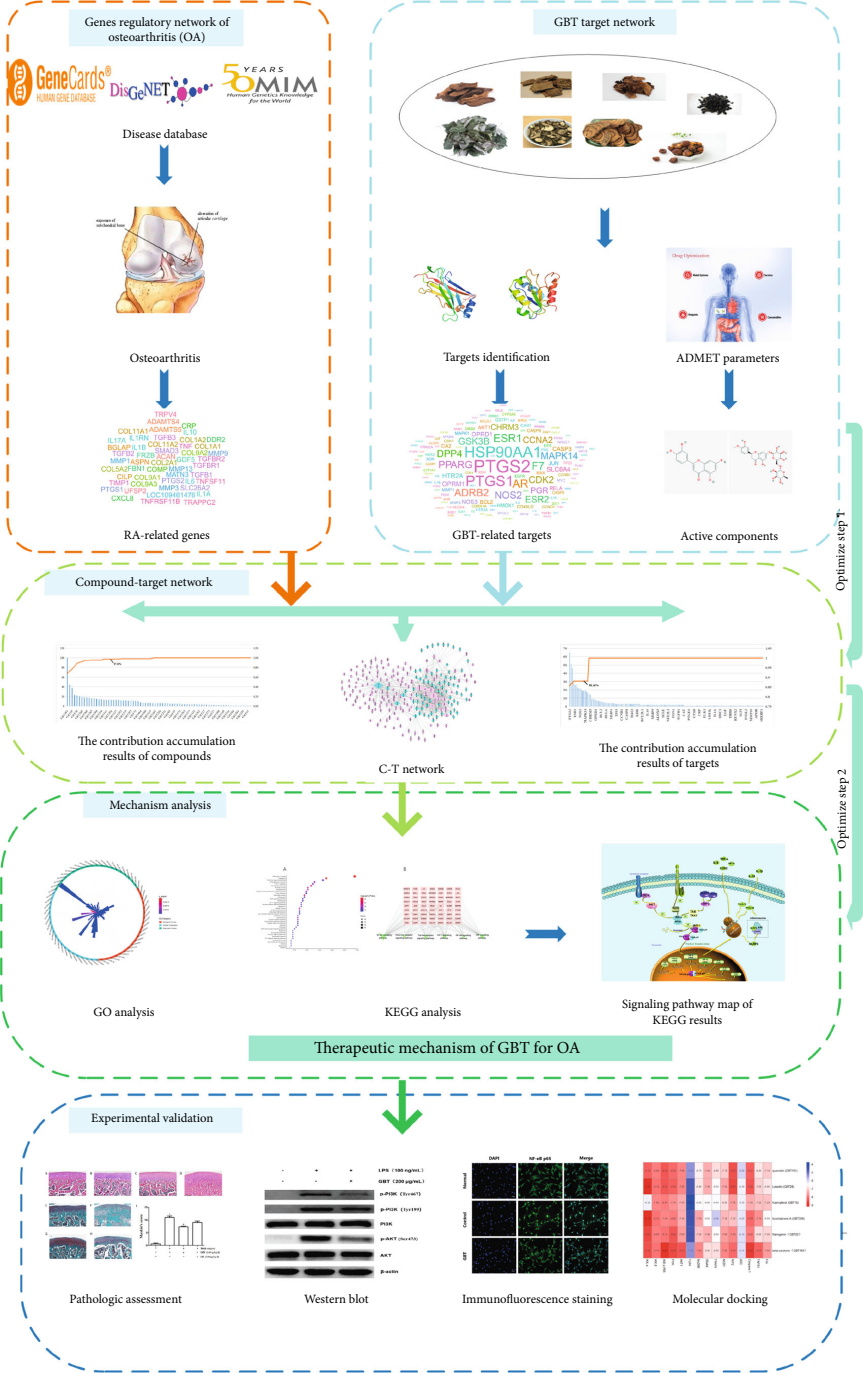
The flowchart of the network pharmacology and experimental validation.

In this study, a network pharmacology module and in vivo and in vitro experiments were used to elucidate the therapeutic mechanisms of GBT for the treatment of OA (Figure 1). Firstly, all GBT compounds were collected from online databases. Next, the active compounds were screened out from all GBT compounds based on the basis of ADME. The targets of these active compounds were identified from public databases. A compound-target (CT) network was established in accordance with connections between active compounds and intersection genes, which were used to further analyze the contribution accumulation results of active compounds and intersection genes. The intersection genes were also analyzed with KEGG and GO enrichment analyses to determine key mechanisms. Finally, the key pathways of GBT were validated with molecular docking, in vivo, and in vitro experiments.

### 3.1. Network Pharmacology Prediction

#### 3.1.1. Key Compounds of GBT

Using a systematic search of compounds in GBT from public databases, a total of 500 compounds were retrieved through TCMSP, YaTCM, TCMID, and Traditional Chinese Medicine Database@Taiwan databases. The number of compounds contained in each botanical drug of GBT predicted with databases is represented on Supplementary Table 1. Every ingredient was given a unique ID, and the detailed information of these compounds is represented on Supplementary Table 2. 87 active compounds were screened out on the basis of absorption, distribution, metabolism, and excretion (ADME), and their corresponding information is presented on Supplementary Table 3.

#### 3.1.2. The Regulatory Network of GBT in the Treatment of OA

A total of 254 targets corresponding to 87 active ingredients of GBT were acquired from TCMSP databases. OA-related genes were recognized from three online databases, 2885 of which were obtained from GeneCards, 368 of which were obtained from DisGeNET, and 102 of which were obtained from OMIM. After eliminating the duplicated genes, 2979 genes were identified. 150 of the intersection genes of GBT-related genes and OA-related genes were obtained, which were defined as therapeutic genes of GBT against OA.

**Figure 2:**
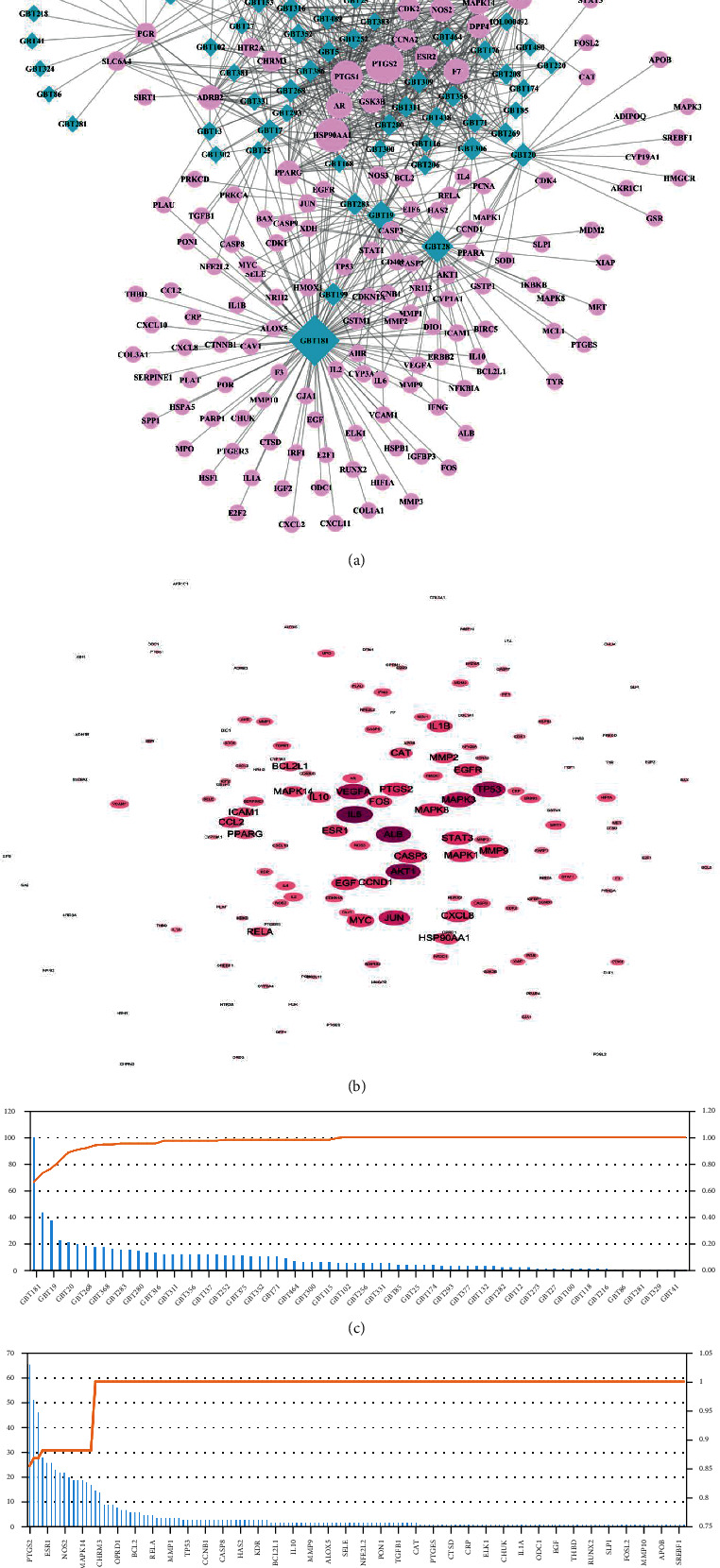
(a) Compound-target network of GBT in the treatment of OA. (b) PPI network of the 150 GBT-OA interaction genes. (c) The contribution accumulation results of the active compounds in the compound-target network. (d) The contribution accumulation results of the targets in the compound-target network.

The PPI network was built to analyze the therapeutic genes of GBT against OA, which showed that IL-6 had the most relative connection with other genes, indicating that it was the most critical gene target in this network. Detailed information of the PPI network is provided in Figure 2(b). Other top 30 ranked hub genes included ALB, AKT1, TP53, VEGFA, MAPK3, JUN, CASP3, STAT3, PTGS2 (COX-2), EGF, MMP9, MAPK1, MYC, CXCL8 (IL-8), CCND1 (BCL1), IL-1*β*, and ESR1. In the onset and progression of OA, these core genes are mainly involved in the expression of inflammatory mediator (IL-6, IL-1*β*, CXCL8, and PTGS2), the activation of inflammatory signaling pathways (AKT1, MAPK3, STAT3, and MAPK1), cartilage matrix degradation (MMP9), synovial neovascularization (VEGFA), and chondrocyte apoptosis (CASP3 and CCND1).

A compound-target (CT) network was established on the basis of connections between active compounds and intersection genes, whose results showed that 78 of the 87 active compounds were involved in the network (Figure 2(a)). Further analysis of the contribution accumulation index of the active compounds suggested that the top 6 compounds including quercetin (GBT181), luteolin (GBT28), kaempferol (GBT19), licochalcone A (GBT306), naringenin (GBT20), and *β*-carotene (GBT199) contribute to 33.3% connective degree with intersection genes (Figure 2(c)). Meanwhile, the results of the analysis also showed that PTGS2 was the intersection genes most closely related to ingredients in GBT, contributing to 9% connections with active compounds (Figure 2(d)).

#### 3.1.3. Pathway Enrichment Analysis

**Figure 3:**
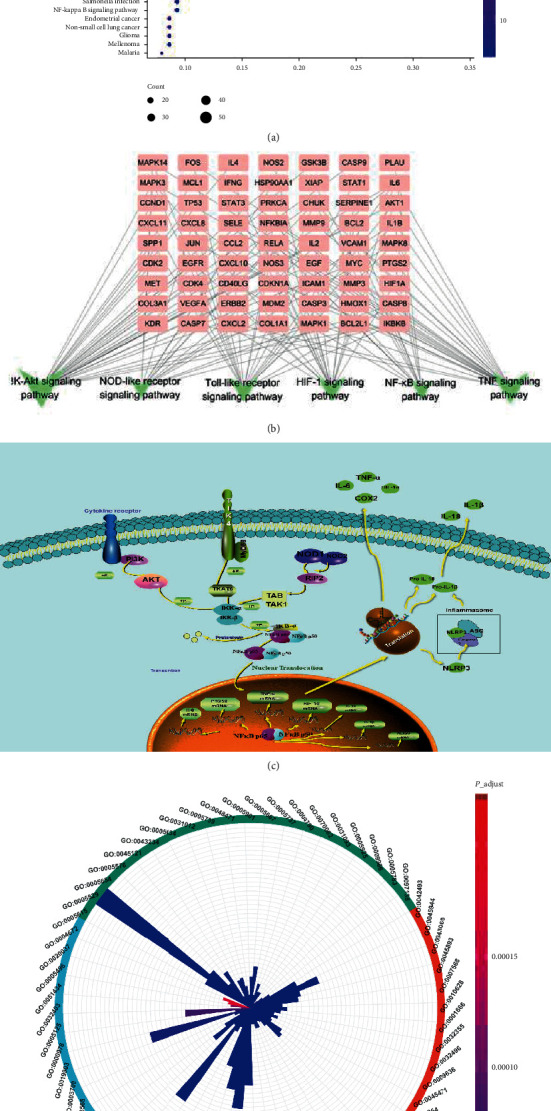
(a) Dot plot for the top 40 KEGG pathways. (b) Network of 6 OA-related pathways identified from KEGG enrichment analysis and their corresponding targets. (c) The associations of signaling pathway predicted by KEGG. (d) GO enrichment analysis.

The GBT-regulating OA genes were analyzed via KEGG enrichment analysis, and pathways satisfying *p* < 0.05 were considered statistically significant. The top 40 pathways of KEGG results were visualized with a dot plot (Figure 3(a)). KEGG results indicated that the PI3K/AKT pathway [35], Toll-like receptor pathway [36], NOD-like receptor pathway [37], TNF pathway [38], and HIF-1 pathway [39] were closely related to OA. A network of 6 OA-related pathways and their corresponding targets is shown in Figure 3(b).

The PI3K/AKT signaling pathway, Toll-like receptor signaling pathway, and NOD-like receptor (NOD1 and NOD2) signaling pathway can induce the ubiquitination and phosphorylation of I*κ*B-*α* after activation and further lead to the degradation of I*κ*B-*α* [40–42]. Degradation of I*κ*B-*α* can relieve the inhibition of the NF*κ*B dimer (composed of NF*κ*B p65 and NF*κ*B p50) and enable it to enter the nucleus and activate the transcription of related inflammatory mediators [43, 44]. Previous studies have shown that the activation of the NF*κ*B signaling pathway can promote the transcription and translation of the NOD-like receptor protein 3 (NLRP3), COX-2, IL-1*β*, IL-6, IL-18, TNF-*α*, and HIF-1*α*. Simultaneously, the formation of inflammasome that NLRP3 is involved in is essential to the activation of the NOD3 pathway, and the inflammasome can activate pro-IL-1*β* and pro-IL-18 and release into the extracellular, promoting further inflammatory response. In addition, TNF-*α* and HIF-1*α* are key to the activation of the TNF and HIF pathways, respectively. In conclusion, the NF*κ*B pathway is the core of the 5 significant KEGG-predicated pathways above. A pathway hypothesis diagram was drawn based on KEGG results (Figure 3(c)).

GO enrichment analysis was performed using the clusterProfiler package of R software to identify the biological functions of the intersection genes with *p* < 0.05. Biological functions were divided into three parts, including biological process, cellular compounds, and molecular function (Figure 3(d)). GO analysis showed that the targets of the intersection genes of GBT-related genes and OA-related genes were enriched in the regulation of transcription (GO:0045944, GO:0045893, GO:0010628, GO:0008134, GO:0004879, GO:0001077, GO:0003700, GO:0019903, and GO:0000978), inflammatory response (GO:0006954, GO:0032355, and GO:0071222), cell proliferation and apoptosis (GO:0043066, GO:0007568, and GO:0008284), regulation of angiogenesis (GO:0001666, GO:0071456, and GO:0045766), and cytokine activity (GO:0005125).

#### 3.1.4. Quality Control of GBT

**Figure 4:**
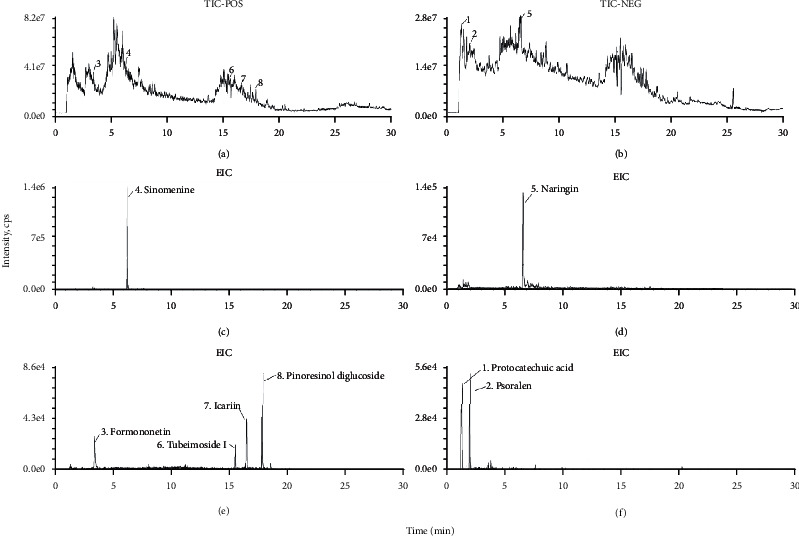
Total ion chromatography (TIC) on positive (a) and negative (b) and extraction ion chromatography (EIC, C-F) of GBT.

The eight core compounds of GBT were collected from the Chinese Pharmacopoeia (2020 Edition) and related literature for quality control [45]. The key compounds of GBT were detected using liquid chromatography coupled with mass spectrometry (LC-MS). The results showed that GBT contain high contents of all eight compounds (Figure 4). Detailed information is presented in Table 4.

### 3.2. In Vivo Experiment Verification

**Figure 5:**
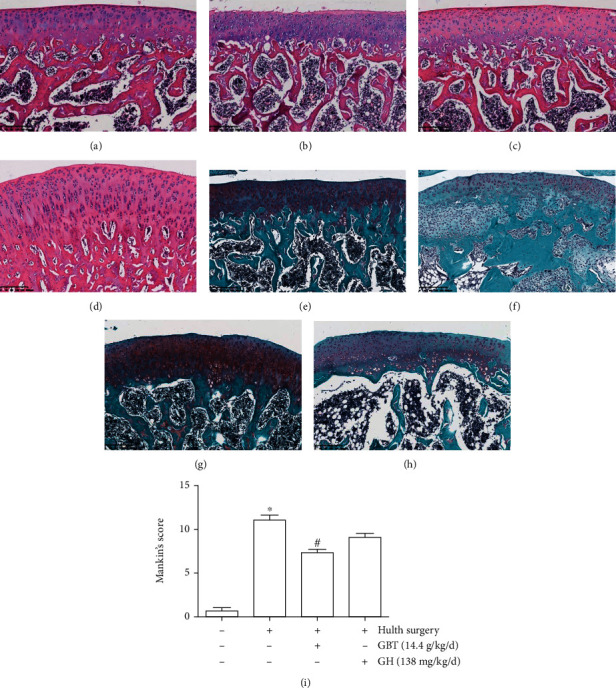
The pathological changes of cartilage. (a) Normal group hematoxylin-eosin staining; (b) OA model group hematoxylin-eosin staining; (c) GBT-treated group hematoxylin-eosin staining; (d) glucosamine hydrochloride intervention group hematoxylin-eosin staining; (e) normal group safranin O/fast green staining; (f) OA model group safranin O/fast green staining; (g) GBT-treated group safranin O/fast green staining; (h) glucosamine hydrochloride intervention group safranin O/fast green staining; (i) Mankin's scoring of each group; the data are expressed as the mean ± standard deviation (^∗^*p* < 0.05 compared with normal group; ^#^*p* < 0.05 compared with OA model group). GBT: Gubitong Recipe; GH: glucosamine hydrochloride.

Safranin O/fast green and hematoxylin-eosin-stained pathological sections of rat knee joints were observed and photographed under a light microscope. Results showed that rats in the normal group had smooth cartilage surfaces. Chondrocytes in the normal group were regularly arranged with a continuous and complete tide line, and the staining was evenly distributed (Figures 5(a) and 5(e)). Rats in the OA model group had rough cartilage surfaces. The arrangement of chondrocytes was irregular, and apoptosis chondrocytes appeared. The number of local chondrocytes increases with an incomplete tide line and lighter matrix staining (Figures 5(b) and 5(f)). Compared with the OA model group, the pathological condition of the GBT group was improved to some extent (Figures 5(c) and 5(g)), while the glucosamine hydrochloride intervention group had no significant improvement (Figures 5(d) and 5(h)). Mankin's score of the GBT group was significantly lower than that of the OA model group, suggesting that GBT can arrest the disease progression of OA (Figure 5(i)).

### 3.3. In Vitro Experiment Verification

#### 3.3.1. Identification of Chondrocytes

**Figure 6:**
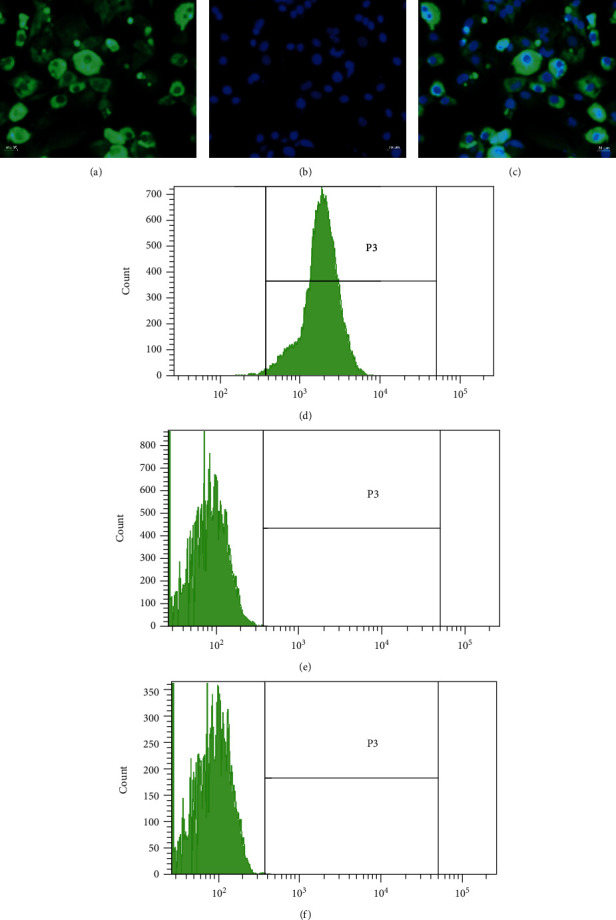
(a) Measurement of type II collagen expression in chondrocytes by immunofluorescent staining. (b) DAPI-labeled nuclei of chondrocytes. (c) Merged image of (a) and (b). (d) The proportion of chondrocytes positive for type II collagen (98.6%). (e) Chondrocytes without the addition of type II collagen antibody as negative control. (f) SW1353 cells as negative control.

Immunofluorescence results showed that chondrocytes isolated from the knee cartilage of suckling rats exhibited strong positive for type II collagen (Figures 6(a)–6(c)). Flow cytometry results suggested that 98.6% of the isolated chondrocytes were positive for type II collagen and SW1353 cells were negative for type II collagen, which indicated that the isolated chondrocytes are of high purity and applicable to subsequent experiments (Figures 6(d)–6(f)).

#### 3.3.2. Regulatory Effect of GBT on Key Targets Predicted by Network Pharmacology

**Figure 7:**
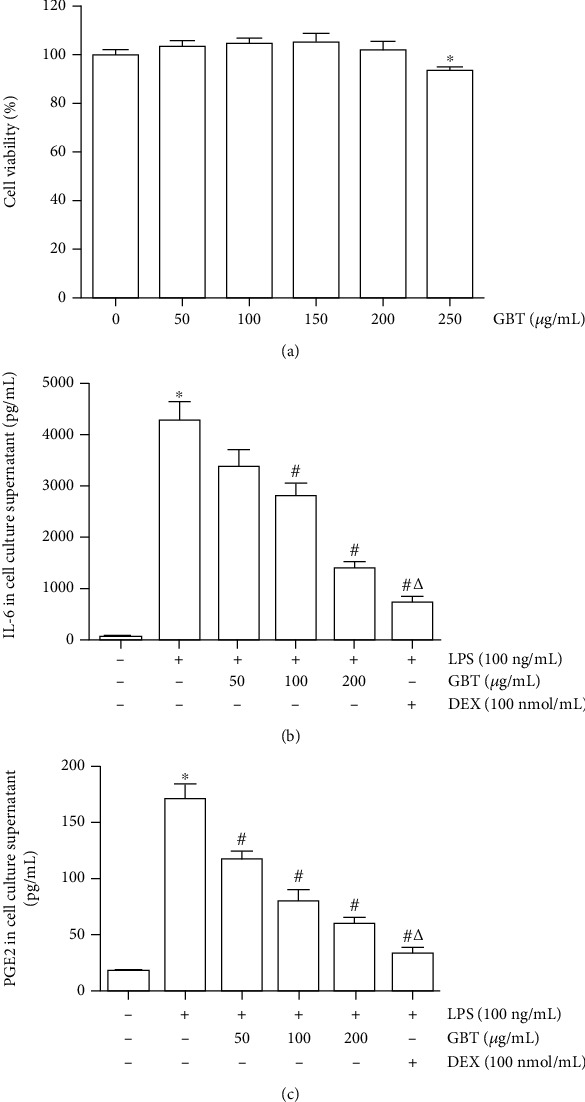
Chondrocytes were pretreated with dexamethasone or different concentrations of GBT for 1 h and stimulated with LPS for 12 h. (a) IL-6 in cell culture supernatant. (b) PGE_2_ in cell culture supernatant. ^∗^*p* < 0.05 compared with blank-control group; ^#^*p* < 0.05 compared with LPS-induced group; ^△^*p* < 0.05 compared with high-dose concentration of GBT group.

After being treated with the GBT solution for 12 h, the cell viability was detected with the MTS assay, whose results showed that 250 *μ*g/mL of the GBT solution remarkably inhibits the cell proliferation in comparison with 200 *μ*g/mL of the GBT solution having no effect. Therefore, 200 *μ*g/mL of the GBT solution is referred to as the high-dose intervention concentration, 100 *μ*g/mL of the GBT solution is considered as the medium-dose intervention concentration, and 50 *μ*g/mL is referred to as the low-dose intervention concentration (Figure 7(a)).

In order to verify the regulatory effect of GBT on the key targets predicted with network pharmacology, LPS, a highly inflammatory endotoxin, was used to stimulate chondrocytes to establish a cellular model of OA. In the above network pharmacology study, IL-6 is the key target predicted via the PPI network. The compound-target network indicated that PTGS2 (COX-2) is the key target of treating OA with GBT, and COX-2 enzymes can catalyze the synthesis of PGE_2_. Also, it has been confirmed by literature that IL-6 and PGE_2_ play a vital role in the occurrence and progression of OA, so IL-6 and PGE_2_ were selected as the characteristic molecules for this research.

50 *μ*g/mL, 100 *μ*g/mL, and 200 *μ*g/mL of GBT were used to intervene the chondrocytes, and 100 *μ*mol/mL of dexamethasone was set as the positive control group. After being pretreated with the above drugs for 1 h, the chondrocytes were stimulated with 100 ng/mL of LPS for 12 h. The contents of PGE_2_ and IL-6 in the supernatant were detected using the ELISA method. The results suggested that LPS could enhance secretion of PGE_2_ and IL-6 in chondrocytes. Pretreatment with GBT and dexamethasone can significantly reduce the contents of PGE_2_ and IL-6 in the supernatant of the LPS-induced chondrocytes, and GBT showed a dose-dependent effect (Figure 7(b)).

#### 3.3.3. Regulatory Effect of GBT on KEGG-Related Pathway

KEGG results suggested that the PI3K/AKT signaling pathway, Toll-like receptor signaling pathway, NOD-like signaling pathway, NF*κ*B signaling pathway, TNF signaling pathway, and HIF-1 signaling pathway were closely related to GBT in treating OA; hence, in vivo experiments for verification were conducted.

**Figure 8:**
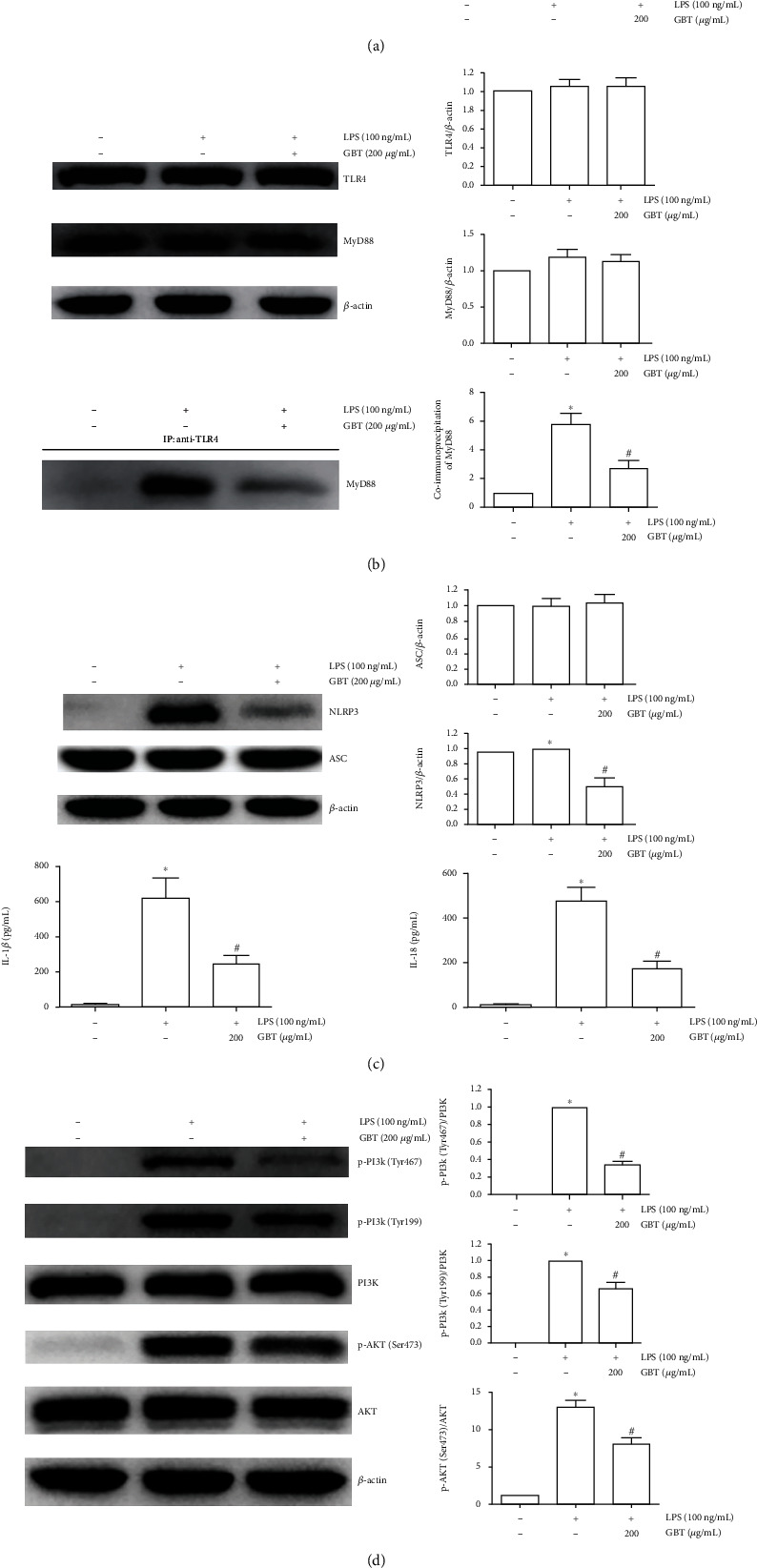
Chondrocytes were pretreated with 200 *μ*g/mL of GBT for 1 h and then stimulated with 100 ng/mL LPS for 30 min or 12 h. (a) The expression of NF*κ*B p65 in the nucleus and I*κ*B-*α* in cells after 30 min of LPS stimulation and immunofluorescence of NF*κ*B p65. (b) TLR4, MyD88, and TLR4-bound MyD88 after 30 min of LPS stimulation. (c) The expression of NLRP3, ASC, IL-18, and IL-1*β* after 12 h of LPS stimulation. (d) PI3K, P-PI3K (Tyr467), P-PI3K (Tyr199), AKT, and p-AKT (Ser473) after 12 h of LPS stimulation. (e) HIF-1*α* expression after 12 h of LPS stimulation. (f) TNF-*α* mRNA expression and TNF-*α* content in cell supernatant after 12 h of LPS stimulation. ^∗^*p* < 0.05 compared with blank-control group; ^#^*p* < 0.05 compared with LPS-induced group.

After being pretreated with 200 *μ*g/mL of GBT for 1 h, the chondrocytes were stimulated with 100 ng/mL of LPS for 30 min. After stimulation with LPS, the content of I*κ*B-*α* in the cytoplasm was significantly decreased, while NF*κ*B p65 in the nucleus increased. The immunofluorescence results of NF*κ*B p65 indicated the transfer of NF*κ*B p65 from the cytoplasm to the nucleus (NF*κ*B nuclear translocation), confirming the activation of the NF*κ*B pathway. Although the contents of TLR4 and MyD88 did not change significantly after LPS stimulation, the coimmunoprecipitation results showed that the content of MyD88 bound to TLR4 increased, suggesting the activation of the TLR4 pathway. In addition, GBT can reverse the change of I*κ*B-*α* in the cytoplasm and NF*κ*B p65 in the nucleus which was induced by LPS and reduce the proportion of the nuclear translocation of NF*κ*B (Figure 8(a)). At the same time, TLR4-bound MyD88 decreased, suggesting that GBT can inhibit the abnormal activation of NF*κ*B and TLR4 pathways (Figure 8(b)).

After being pretreated with 200 *μ*g/mL of GBT for 1 h, the chondrocytes were stimulated with 100 ng/mL LPS for 12 h. We detected the expression of the NOD-like receptor protein 3 (NLRP3) and ASC, the key protein in the NOD pathway, and the contents of IL-1*β* and IL-18 in the cell culture supernatant, which were the downstream inflammatory factors regulated by the NLRP3 inflammasome. Results showed that the expressions of NLRP3, IL-1*β*, and IL-18 increased after LPS stimulation. 200 *μ*g/mL of GBT could reverse the above changes, but the content of ASC was not affected by LPS and GBT (Figure 8(c)). After LPS stimulation, the protein contents of PI3K and AKT in the PI3K/AKT pathway did not change significantly, but the phosphorylation level increased significantly, suggesting abnormal activation of the PI3K/AKT pathway. 200 *μ*g/mL of GBT can reverse the increase of PI3K and AKT phosphorylation induced by LPS to a certain extent (Figure 8(d)). The production of HIF-1*α* and TNF-*α* is regulated by NF*κ*B, which is a key protein to activate the HIF pathway and TNF pathway. After LPS stimulation, the protein level of HIF-1*α* and the mRNA expression and protein level in the supernatant of TNF-*α* were significantly increased, which can be reversed with 200 *μ*g/mL of GBT (Figures 8(e) and 8(f)).

### 3.4. Molecular Docking of Active Compound and Key Target

Molecular docking was conducted with AutoDockTools to assess the binding energy between key compounds from GBT and core proteins of key pathways predicted by KEGG analysis. Core proteins include PI3K (PDB ID:1E7V) [46] of PI3K/AKT pathway, TLR4 (PDB ID:5NAO) [47], MyD88 (PDB ID:2Z5V) [48], TRAF6 (PDB ID:1LB4) [49] of the Toll-like receptor pathway, RIP2 (PDB ID:5NG3) [50], ASC (PDB ID:1UCP) [51], caspase-1 (PDB ID:5FNA) [52] of the NOD-like receptor pathway, IKK-*α* (PDB ID:5TQW) [53], IKK-*β* (PDB ID:4KIK) [54], I*κ*B-*α*/NF*κ*B p65 (PDB ID:1K3Z) [55] of the NF*κ*B pathway, TNFR1 (PDB ID:7K7A) [56] of the TNF pathway, and HIF-1*α* inhibitor (PDB ID:4Z1V) [57] of the HIF-1 pathway.

**Figure 9:**
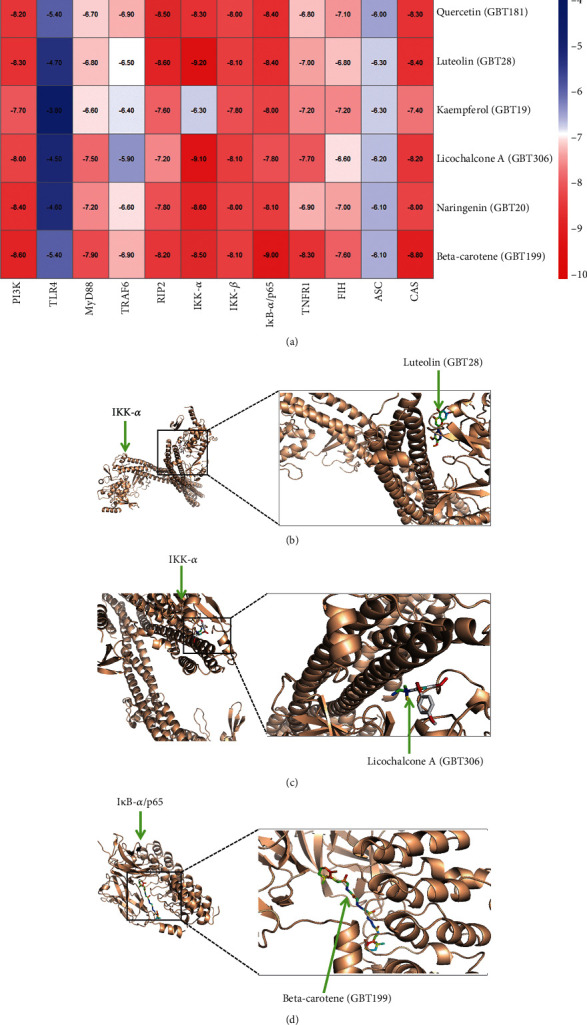
(a) Heat map of binding energy between key compounds from GBT and key proteins in signal pathways. (b) Binding site of luteolin with IKK-*α*. (b) Binding site of licochalcone A with IKK-*α*. (c) Binding site of *β*-carotene with I*κ*B-*α*/NF*κ*B p65.

In this study, quercetin (GBT181), luteolin (GBT28), kaempferol (GBT19), licochalcone A (GBT306), naringenin (GBT20), and *β*-carotene (GBT199) were selected as key active compounds in accordance with the compound-target network result. Proteins that possess crucial roles in the PI3K/AKT pathway, Toll-like receptor pathway, NOD-like receptor pathway, TNF pathway, and HIF-1 pathway were chosen as key targets. The binding affinity (KD) was calculated using Vina software, and subsequently, a heat map was constructed (Figure 9(a)). The result of molecular docking showed that three groups, including luteolin and IKK-*α*, licochalcone A and IKK-*α*, *β*-carotene and I*κ*B-*α*/NF*κ*B p65, had binding affinities larger than 9.0, indicating they had strong bonding abilities with each other. The binding modes visualized by PyMOL for the above ligands and receptors are illustrated in Figures 9(b)–9(d).

### 3.5. Verification of Key Mechanism by Blocking NF*κ*B Pathway

Literature research showed that the PI3K/AKT pathway, NOD-like pathway, and Toll-like pathway activated the NF*κ*B pathway through signal transduction. The activated NF*κ*B pathway then affected the HIF pathway and TNF pathway through a transcriptional regulator. The above process suggested that the NF*κ*B pathway is the key to GBT in the treatment of OA. The supposition was further strengthened by molecular docking, which showed that the key molecules in GBT had higher binding energy by combining with the key nodes in NF*κ*B.

**Figure 10:**
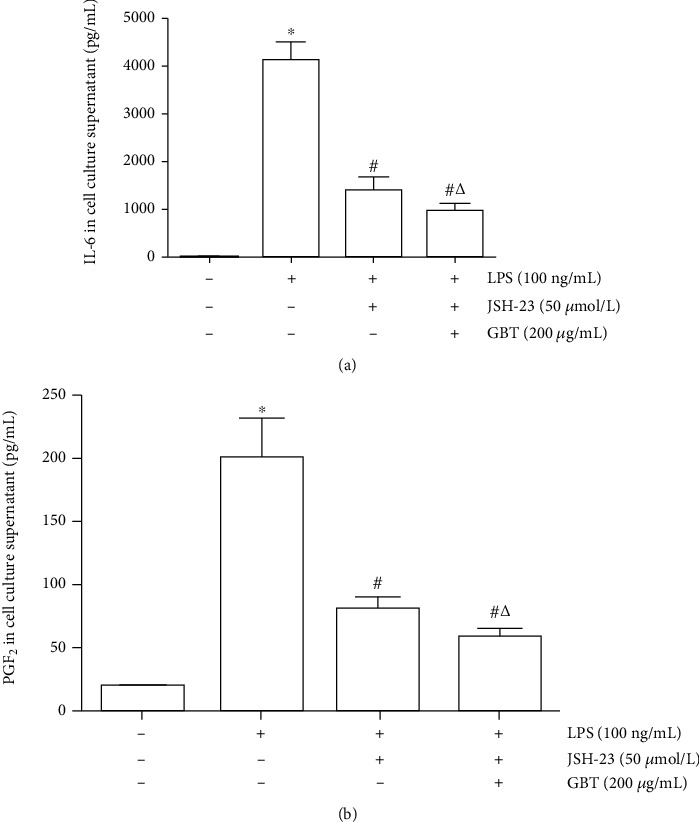
Chondrocytes were pretreated with JSH-23 (with or without GBT) for 1 h and stimulated with LPS for 12 h. (a) IL-6 in cell culture supernatant. (b) PGE_2_ in cell culture supernatant. ^∗^*p* < 0.05 compared with blank-control group; ^#^*p* < 0.05 compared with LPS-induced group; ^△^*p* > 0.05 compared with JSH-23-treated group.

Hence, the NF*κ*B pathway was blocked to verify the significant roles of NF*κ*B in the treatment of OA with GBT. JSH-23, a blocker that specifically blocked the nuclear translocation of NF*κ*B, has been used in our previous experiments. Results showed that JSH-23 could significantly inhibit LPS-induced IL-6 and PGE_2_ expression. The concomitant use of JSH-23 and GBT was not superior to the use of JSH-23 alone, confirming that the NF*κ*B pathway is the key to the inhibitory effects of GBT on the inflammatory reaction of chondrocytes (Figure 10).

## 4. Discussion

In our previous RCT study, eight weeks of orally administrated GBT significantly relieved OA-induced pain and joint stiffness and improved the quality of life, and the efficacy was superior to that of glucosamine hydrochloride [6]. In this study, the pathological sections of the knee joint demonstrated that GBT had a good protective effect on the OA model cartilage established via Hulth surgery.

Chronic inflammatory response plays an important role in the occurrence and progressive stages of OA. Joint trauma, senescence of chondrocytes, and inflammatory mediators induced by other inflammatory joint diseases (such as rheumatoid arthritis and psoriatic arthritis) are all triggers for chondrocytes to initiate inflammatory response [58]. Inflammatory response was rapidly induced by inflammatory factors, resulting in overexpression and release of cartilage matrix proteolytic enzymes such as MMPs and ADAMSTS, and ultimately destroyed the balance of synthesis and metabolism of the cartilage matrix [59]. The inflammatory response of chondrocytes is regulated by a complex regulatory network of inflammatory mediators, among which IL-6, TNF-*α*, IL-1*β*, IL-18, and PGE_2_ play a significant role in the development of OA [60]. The expression of IL-6, TNF-*α*, IL-1*β*, IL-18, and PGE_2_ increased after stimulation of chondrocytes with inflammatory factors in vitro [61, 62]. In clinical practice, NSAIDs and selective COX-2 inhibitors have been used as first-line drugs in the treatment of OA, whose mechanism is to reduce the synthesis of PGE_2_ by inhibiting COX-2, thus exerting anti-inflammatory, pain-relieving, and joint-protective effects [63]. In addition, inhibition of IL-6 and IL-1*β* can effectively relieve the symptoms of OA [64, 65].

In this study, network pharmacology revealed that PTGS2 (COX-2) is a key gene in the process of OA being treated with GBT, and IL-6 is the core protein regulated by these genes. Since COX-2 regulates the catalytic synthesis of PGE_2_, PGE_2_ and IL-6 were predicted to be the core molecules of GBT in the treatment of OA in this study. The prediction was further verified through in vitro experiments. After stimulating chondrocytes with LPS, the secretion of PGE_2_ and IL-6 increased significantly, while GBT could inhibit the expression of these compounds and showed a dose-dependent effect. It follows from the above results that the anti-inflammatory effect of GBT relies on its inhibitory effect on key OA-related inflammatory factors.

In the subsequent research on the predicted pathway, after stimulation of chondrocytes with LPS, I*κ*B-*α* in the cytoplasm was reduced and NF*κ*B p65 in the nucleus was increased. The immunofluorescence assay also indicated that NF*κ*B p65 was transferred from the cytoplasm to the nucleus, suggesting the activation of the NF*κ*B pathway [66]. After GBT intervention, the NF*κ*B pathway was significantly inhibited, and the expression of TNF-*α* and HIF-1*α* regulated by NF*κ*B significantly decreased.

The increase of p-PI3K and p-AKT indicated that the PI3K/AKT pathway was aberrantly activated [67]. Although the total amount of TLR4 and MyD88 was not significantly changed, the quantity of MyD88 binding with TLR4 was significantly increased, indicating that the Toll-like receptor signaling pathway was activated [68]. Activation of the inflammatory response through the formation of the NOD-like receptor protein 3 (NLRP3) inflammasome is one of the classical NOD pathways, which increased secretion of the NLRP3 protein and downstream IL-18 and IL-1*β* suggesting the abnormal activation of the NLRP3 pathway by LPS [69]. GBT can effectively inhibit the expression of p-PI3K, p-AKT, NLRP3, IL-18, and IL-1*β* and reduce the amount of MyD88 binding with TLR4, suggesting that the PI3K/AKT signaling pathway, Toll-like receptor 4 signaling pathway, and NLRP3 signaling pathway aberrantly activated by LPS are inhibited.

Molecular docking is an in silico approach to predict and evaluate the binding affinity of an active compound against a ligand. This study utilized the method to predict key compounds of GBT and core proteins of predicted pathways [70–73]. In order to further confirm the potential mechanism of the GBT-related compounds, molecular docking was conducted between the key active compounds and the key nodes in the KEGG-related signaling pathway. Results indicated that luteolin and IKK-*α*, licochalcone A and IKK-*α*, *β*-carotene and I*κ*B-*α*/NF*κ*B p65 had a higher binding energy, suggesting that the NF*κ*B pathway might be the critical mechanism to the process of OA being treated with GBT.

Therefore, the NF*κ*B pathway was blocked using a specific blocker in further experiments and subsequently intervened using GBT. The results showed that after blocking the NF*κ*B pathway, the effect of GBT on the key targets PGE_2_ and IL-6 was significantly diminished. The above results suggested a crucial role of the NF*κ*B signaling pathway in the treatment of OA with GBT.

In this study, we confirmed the multitarget effect of GBT in the treatment of OA through network pharmacological prediction and experimental verification. GBT can inhibit several inflammation-related signaling pathways and ultimately reduce the expression of inflammation-related mediators, indicating a key role of anti-inflammatory effect of GBT in the treatment of OA. Previous studies have shown that cartilage inflammatory response is an essential agent of joint pain and stiffness in OA patients; suppression of this inflammatory response alleviates these symptoms, which supports the discovery that GBT exerts an anti-inflammatory effect on OA in this study [74]. Further studies will be conducted in animal experiments to investigate how GBT exerts a protective effect on the cartilage matrix by inhibiting the inflammatory response.

At the same time, we identified core compounds in the treatment of OA with GBT through network pharmacology including quercetin, luteolin, kaempferol, licochalcone A, naringenin, and *β*-carotene. A thorough review of the literature revealed that these ingredients are usually insoluble in water. In clinical applications, we usually decoct botanical drugs to produce an oral solution of GBT, which may perhaps cause excessive loss of key components. In future experiments, we may be able to optimize the extraction method and produce an oral solution of larger key ingredient contents so as to promote clinical efficacy.

## Figures and Tables

**Table 1:** Prime sequences for quantitative real-time PCR.

Gene name	Sequence (5′ to 3′)
Rat IL-6 sense	ACCACCCACAACAGACCAGT
Rat IL-6 antisense	ACAGTGCATCATTCGCTGTTC
Rat TNF-*α* sense	AAATGGGCTCCCTCTCATCAGTTC
Rat TNF-*α* antisense	TCTGCTTGGTGGTTTGCTACGAC
Rat HIF-*α* sense	GCTGCCTCTTCGACAAGCTT
Rat HIF-*α* antisense	CGCTGGAGCTAGCAGAGTCA
Rat *β*-actin sense	CACCCGCGAGTACAACCTTC
Rat *β*-actin antisense	CCCATACCCACCATCACACC

**Table 2:** Mankin's score evaluation.

*Cartilage structure*	
Normal volume, smooth surface	0
Irregular cartilage surface	1
Irregular cartilage surface and pannus formation	2
Fissures to transfer layer	3
Fissures to radiation layer	4
Fissures to cartilage matrix calcification layer	5
Complete destruction of cartilage structure	6
*Chondrocytes*	
Normal	0
Diffuse cytosis	1
Proliferative chondrocyte clusters	2
Decreased cell number	3
*Cartilage matrix (safranin O) staining*	
Normal	0
Slightly weakened	1
Moderately weakened	2
Strongly weakened	3
No color change	4
*Tide line*	
Complete and continuous	0
Noncontinuous and is crossed by blood vessels	1
*Total*	14

**Table 3:** Information and dilution rates of antibodies.

Name	Manufacture	Item no.	Dilution rate
AKT antibody	Cell Signaling Technology	4691S	1 : 1000
p-AKT (Ser473) antibody	Cell Signaling Technology	4060S	1 : 1000
NF*κ*B p65 antibody	Cell Signaling Technology	8242 T	1 : 1000
I*κ*B-*α* antibody	Cell Signaling Technology	4814 T	1 : 800
Lamin B1 antibody	ABclonal	A11495	1 : 1500
NLRP3 antibody	ABclonal	A14233	1 : 1000
TLR4 antibody	ABclonal	A17436	1 : 1000
MyD88 antibody	ABclonal	A0786	1 : 800
PI3K p85 antibody	ABclonal	A4992	1 : 500
HIF-1*α*	ImmunoWay	YT2133	1 : 1000
p-PI3K (Y467/199)	ImmunoWay	YP0224	1 : 1000
*β*-actin antibody	Zhongshan Jinqiao	TA-09	1 : 1000
HRP-conjugated goat antimouse IgG	Zhongshan Jinqiao	ZB-5305	1 : 5000
HRP-conjugated goat antirabbit IgG	Zhongshan Jinqiao	ZB-2301	1 : 5000

**Table 4:** Chemical identification of Gubitong prescription.

No.	RT (min)	Name	Formula	Ion	Cal. (m/z)	Mea. (m/z)	Error (ppm)	MS/MS
1	1.21	Protocatechuic acid	C_7_H_6_O_4_	M-H	153.0193	153.0188	4.385	153.0188, 109.0288
2	1.98	Psoralen	C_11_H_6_O_3_	M-H	185.0244	185.0246	0..786	185.0246, 147.0325
3	3.37	Formononetin	C_16_H_12_O_4_	M+H	269.0808	269.0812	3.337	269.0812
4	6.20	Sinomenine	C_19_H_23_NO_4_	M+H	330.1699	330.1701	2.745	330.1701
5	6.71	Naringin	C_27_H_32_O_14_	M-H	579.1719	579.171	0.33	579.171, 425.1429
6	15.45	Tubeimoside I	C_63_H_98_O_29_	M+H	1319.6266	1319.627	2.759	1319.627
7	16.39	Icariin	C_33_H_40_O_15_	M+H	677.2439	677.2445	2.827	677.2442, 369.1445, 313.0728
8	17.78	Pinoresinol diglucoside	C_32_H_42_O_16_	M+H	683.2545	683.2536	-2.695	519.1936, 357.2159

## Data Availability

The data used to support the findings of this study are included within the manuscript and Supplementary Materials.

## Supplementary Materials

Supplementary Materials Supplementary Table 1: the number of compounds contained in each botanical drug. Supplementary Table 2: the detailed information of compounds contained in GBT. Supplementary Table 3: the information of active compounds in GBT.
