# Supplementary material for: Integrating Network Pharmacology and Experimental Validation to Explore the Key Mechanism of Gubitong Recipe in the Treatment of Osteoarthritis
Source: Comput Math Methods Med. 2022 Jun 8;2022:7858925. doi: 10.1155/2022/7858925 (PMC9200584; doi:10.1155/2022/7858925)
Supplement: Supplementary Materials — Supplementary Table 1: the number of compounds contained in each botanical drug. Supplementary Table 2: the detailed information of compounds contained in GBT. Supplementary Table 3: the information of active compounds in GBT. [file 7858925.f1.zip › Supplymentary Table 1 (1).docx]

**Supplymentary Table 1** The number of compounds contained in each herb

| Herbs | Compounds |
| --- | --- |
| *Drynaria fortunei* (Kunze) J.Sm. | 71 |
| *Epimedium brevicomu* Maxim. | 130 |
| *Psoralea corylifolia* L. | 39 |
| *Eucommia u1moides* Oliv. | 147 |
| *Cibotium barometz* (L.) J.Sm. | 34 |
| *Bolbostemma paniculatum* (Maxim.) Franquet. | 46 |
| *Sinomenium acutum* (Thunb.) Rehd. et Wils. | 16 |
| *Spatholobus suberectus* Dunn. | 68 |
| Total | 500 |
