# Supplementary material for: Integrating Network Pharmacology and Experimental Validation to Explore the Key Mechanism of Gubitong Recipe in the Treatment of Osteoarthritis
Source: Comput Math Methods Med. 2022 Jun 8;2022:7858925. doi: 10.1155/2022/7858925 (PMC9200584; doi:10.1155/2022/7858925)
Supplement: Supplementary Materials — Supplementary Table 1: the number of compounds contained in each botanical drug. Supplementary Table 2: the detailed information of compounds contained in GBT. Supplementary Table 3: the information of active compounds in GBT. [file 7858925.f1.zip › Supplymentary Table 2 (1).docx]

**Supplymentary Table 2** The detailed information of compounds contained in GBT

| **ID** | **Name** | **MW** |
| --- | --- | --- |
| GBT1 | (2R)-5,7-dihydroxy-2-(4-hydroxyphenyl)chroman-4-one | 272.27 |
| GBT2 | protocatechuic acid | 154.13 |
| GBT3 | Pinoresinol | 358.42 |
| GBT4 | Secoisolariciresinol | 362.46 |
| GBT5 | Aureusidin | 286.25 |
| GBT6 | sinapic acid | 224.23 |
| GBT7 | cinnamic acid | 148.17 |
| GBT8 | Hopene | 410.8 |
| GBT9 | Narirutin | 580.59 |
| GBT10 | Diplopterol | 428.82 |
| GBT11 | Campesteryl ferulate | 576.94 |
| GBT12 | Eriodyctiol (flavanone) | 288.27 |
| GBT13 | Stigmasterol | 412.77 |
| GBT14 | Dianthoside | 288.28 |
| GBT15 | Vetol | 126.12 |
| GBT16 | Sitogluside | 576.95 |
| GBT17 | beta-sitosterol | 414.79 |
| GBT18 | orientin | 448.41 |
| GBT19 | kaempferol | 286.25 |
| GBT20 | naringenin | 272.27 |
| GBT21 | Afzelin | 432.41 |
| GBT22 | Chinoinin | 422.37 |
| GBT23 | Isomangiferin | 422.37 |
| GBT24 | (+)-catechin | 290.29 |
| GBT25 | eriodictyol | 288.27 |
| GBT26 | Astragalin | 448.41 |
| GBT27 | digallate | 322.24 |
| GBT28 | luteolin | 286.25 |
| GBT29 | darutoside | 574.93 |
| GBT30 | (-)-epicatechin | 290.29 |
| GBT31 | Kushenol I | 438.51 |
| GBT32 | leachianone,a | 438.56 |
| GBT33 | epi-Afzelechin | 274.29 |
| GBT34 | procyanidin b2 | 578.56 |
| GBT35 | HMF | 126.12 |
| GBT36 | p-coumaric acid | 164.17 |
| GBT37 | luteolin-7-o-glucoside | 448.41 |
| GBT38 | (+)-Pinoresinol-.beta.-D-glucoside | 520.58 |
| GBT39 | Threo-dihydroxydehydrodiconiferyl alcohol | 392.44 |
| GBT40 | (+)-catechin-5-O-β-D-glucopyranoside | 452.45 |
| GBT41 | 22-Stigmasten-3-one | 412.77 |
| GBT42 | Aureusidin-6-glucoside | 448.41 |
| GBT43 | Cyclolaudenol acetate | 482.87 |
| GBT44 | Epiafzelechin 5-O-beta-D-glucopyranoside | 436.45 |
| GBT45 | (2R,3R,4R,5S,6R)-2-[(2R,3R)-2-(3,4-dihydroxyphenyl)-5,7-dihydroxy-chroman-3-yl]oxy-6-(hydroxymethyl)tetrahydropyran-3,4,5-triol | 452.45 |
| GBT46 | Eriodictyol 7-glucuronide | 464.41 |
| GBT47 | Isoglaucanone | 384.71 |
| GBT48 | Kaempferol 7-alpha-L-arabinoside | 418.38 |
| GBT49 | Lucenin-2 | 610.57 |
| GBT50 | Luteolin 7-O-glucuronide | 462.39 |
| GBT51 | Luteolin 7-glucoside-4'-neohesperidoside | 756.73 |
| GBT52 | Prunin | 434.43 |
| GBT53 | Procyanidin D | 578.56 |
| GBT54 | Chitranone | 374.36 |
| GBT55 | cycloartenone | 424.78 |
| GBT56 | cyclolaudenol | 440.83 |
| GBT57 | davallioside A | 535.55 |
| GBT58 | davallioside A_qt | 373.39 |
| GBT59 | davallioside B | 535.55 |
| GBT60 | davallioside B_qt | 373.39 |
| GBT61 | eriodictyol-7-O-glucoside | 450.43 |
| GBT62 | Fernene | 410.8 |
| GBT63 | ferna-7-9(11)-diene | 408.78 |
| GBT64 | (4-hydroxyphenyl)-(2,4,6-trihydroxyphenyl)methanone | 246.23 |
| GBT65 | (2S)-2-(2,4-dihydroxyphenyl)-7-hydroxy-8-[(2R)-2-isopropenyl-5-methylhex-4-enyl]-5-methoxy-4-chromanone | 438.56 |
| GBT66 | marioside | 458.56 |
| GBT67 | marioside_qt | 296.4 |
| GBT68 | Neoeriocitrin | 596.59 |
| GBT69 | procyanidin B-5 | 594.56 |
| GBT70 | Vexibinol | 424.53 |
| GBT71 | xanthogalenol | 354.43 |
| GBT72 | PYG | 126.12 |
| GBT73 | vanillic acid | 168.16 |
| GBT74 | EIC | 280.5 |
| GBT75 | LOLIOLIDE | 196.27 |
| GBT76 | Trochol | 442.8 |
| GBT77 | genipin | 226.25 |
| GBT78 | Geniposidic acid | 374.38 |
| GBT79 | geniposidie acid_qt | 212.22 |
| GBT80 | MAE | 116.08 |
| GBT81 | Tar | 150.1 |
| GBT82 | Sinapyl alcohol | 210.25 |
| GBT83 | Heriguard | 354.34 |
| GBT84 | Ferulaldehyde | 178.2 |
| GBT85 | 40957-99-1 | 388.45 |
| GBT86 | Mairin | 456.78 |
| GBT87 | caffeic acid | 180.17 |
| GBT88 | Skimmetin | 162.15 |
| GBT89 | Coniferin | 342.38 |
| GBT90 | Coniferol | 180.22 |
| GBT91 | HYKOP | 182.19 |
| GBT92 | Dunnisinin | 226.25 |
| GBT93 | (-)-Olivir | 376.44 |
| GBT94 | Urushiol III | 316.53 |
| GBT95 | Syrigin | 372.41 |
| GBT96 | (+)-Syringaresinol | 418.48 |
| GBT97 | cis-p-Coumarate | 164.17 |
| GBT98 | Cinnamic acid, 3,4-dimethoxy- (8CI) | 208.23 |
| GBT99 | rutin | 610.57 |
| GBT100 | olivil | 376.44 |
| GBT101 | Hirsutrin | 464.41 |
| GBT102 | Erythraline | 297.38 |
| GBT103 | geniposide | 388.41 |
| GBT104 | genistein | 270.25 |
| GBT105 | 11-deoxyglycyrrhetic acid | 456.78 |
| GBT106 | ursolic acid | 456.78 |
| GBT107 | Nonacosanediol-6,8 | 188.35 |
| GBT108 | Vulgarin | 264.35 |
| GBT109 | Ombuin | 330.31 |
| GBT110 | Acanthoside B | 580.64 |
| GBT111 | 5-o-caffeoylquinic acid | 354.34 |
| GBT112 | Montanic acid | 424.84 |
| GBT113 | (2S,3R,4S,5S,6R)-2-[4-[(1R,3aR,4S,6aS)-6a-hydroxy-4-(4-hydroxy-3-methoxyphenyl)-3,3a,4,6-tetrahydro-1H-furo[4,3-c]furan-1-yl]-2-methoxyphenoxy]-6-(hydroxymethyl)oxane-3,4,5-triol | 536.58 |
| GBT114 | AIDS214634 | 374.42 |
| GBT115 | 3-beta-Hydroxymethyllenetanshiquinone | 294.32 |
| GBT116 | ent-Epicatechin | 290.29 |
| GBT117 | (+)-Cycloolivil | 376.44 |
| GBT118 | Yangambin | 446.54 |
| GBT119 | [(1S,4aS,5R,7S,7aS)-4a,5-dihydroxy-7-methyl-1-[(2S,3R,4S,5S,6R)-3,4,5-trihydroxy-6-(hydroxymethyl)oxan-2-yl]oxy-1,5,6,7a-tetrahydrocyclopenta[c]pyran-7-yl] ethanoate | 406.43 |
| GBT120 | Harpagide 7-acetate_qt | 244.27 |
| GBT121 | reptoside | 390.43 |
| GBT122 | reptoside_qt | 228.27 |
| GBT123 | 6,6'-Dimethoxygossypol | 550.7 |
| GBT124 | 16-Triacontanol | 438.92 |
| GBT125 | catechol | 110.12 |
| GBT126 | (2S,3R,4S,5S,6R)-2-[4-[(1R,3aR,4S,6aS)-6a-hydroxy-4-[3-methoxy-4-[(2S,3R,4S,5S,6R)-3,4,5-trihydroxy-6-(hydroxymethyl)oxan-2-yl]oxyphenyl]-3,3a,4,6-tetrahydro-1H-furo[4,3-c]furan-1-yl]-2-methoxyphenoxy]-6-(hydroxymethyl)oxane-3,4,5-triol | 698.74 |
| GBT127 | (2S,3R,4S,5S,6R)-2-[4-[(1S,3aS,4R,6aR)-3a-hydroxy-4-(4-hydroxy-3-methoxyphenyl)-3,4,6,6a-tetrahydro-1H-furo[4,3-c]furan-1-yl]-2-methoxyphenoxy]-6-(hydroxymethyl)oxane-3,4,5-triol | 536.58 |
| GBT128 | (+-)-Threo-guaiacylglycerol | 214.24 |
| GBT129 | (4aS,8aR)-3-isopropylidene-8a-methyl-5-methylene-2-decalinone | 218.37 |
| GBT130 | Eucommin A | 550.61 |
| GBT131 | (+)-Medioresinol di-O-beta-D-glucopyranoside | 712.77 |
| GBT132 | (+)-medioresinol | 388.45 |
| GBT133 | (+)-Pinoresinol-di-O-β-D-glucoside | 682.74 |
| GBT134 | (2S,3R,4S,5S,6R)-2-[4-[[(3S,4R,5S)-3-hydroxy-5-(4-hydroxy-3-methoxyphenyl)-4-(hydroxymethyl)oxolan-3-yl]methyl]-2-methoxyphenoxy]-6-(hydroxymethyl)oxane-3,4,5-triol | 538.6 |
| GBT135 | (2S,3R,4S,5S,6R)-2-[4-[(2S,3R,4S)-4-hydroxy-3-(hydroxymethyl)-4-[[3-methoxy-4-[(2S,3R,4S,5S,6R)-3,4,5-trihydroxy-6-(hydroxymethyl)oxan-2-yl]oxyphenyl]methyl]oxolan-2-yl]-2-methoxyphenoxy]-6-(hydroxymethyl)oxane-3,4,5-triol | 700.76 |
| GBT136 | (2S,3R,4S,5S,6R)-2-[4-[(2S,3R,4S)-4-hydroxy-4-[(4-hydroxy-3-methoxyphenyl)methyl]-3-(hydroxymethyl)oxolan-2-yl]-2-methoxyphenoxy]-6-(hydroxymethyl)oxane-3,4,5-triol | 538.6 |
| GBT137 | (-)-Tabernemontanine | 354.49 |
| GBT138 | 1-Deoxyeucommiol | 172.25 |
| GBT139 | 3-Furyl-2-ethylacrolein | 150.19 |
| GBT140 | MHP | 166.19 |
| GBT141 | (2R,3R)-3-hydroxyproline | 131.15 |
| GBT142 | [(1S,4aR,5S,7S,7aS)-5-hydroxy-7-methyl-1-[(2S,3R,4S,5S,6R)-3,4,5-trihydroxy-6-(hydroxymethyl)oxan-2-yl]oxy-4a,5,6,7a-tetrahydro-1H-cyclopenta[c]pyran-7-yl] ethanoate | 390.43 |
| GBT143 | Ajugoside_qt | 228.27 |
| GBT144 | aucubin | 346.37 |
| GBT145 | Aucubin_qt | 184.21 |
| GBT146 | Chlorogenin | 432.71 |
| GBT147 | 3-[4-(1H-indol-3-yl)-3H-thiazol-2-ylidene]indole | 315.42 |
| GBT148 | Civetone | 250.47 |
| GBT149 | Cyclopamine | 411.69 |
| GBT150 | Dehydrodiconiferyl alcohol 4,gamma'-di-O-beta-D-glucopyanoside | 682.74 |
| GBT151 | Dehydrodiconiferyl alcohol 4,gamma'-di-O-beta-D-glucopyanoside_qt | 358.42 |
| GBT152 | Dehydrodieugenol | 326.42 |
| GBT153 | Cinchonan-9-al, 6'-methoxy-, (9R)- | 324.46 |
| GBT154 | (2R,3S)-2-amino-3-hydroxy-succinic acid | 149.12 |
| GBT155 | Eucommiol | 188.25 |
| GBT156 | Eucommioside-II | 350.41 |
| GBT157 | Eucommioside | 350.41 |
| GBT158 | Genioisidic acid | 374.38 |
| GBT159 | GBGB | 550.57 |
| GBT160 | ZINC00394284 | 198.24 |
| GBT161 | harpagoside | 494.54 |
| GBT162 | Harpagoside_qt | 332.38 |
| GBT163 | Helenalin | 262.33 |
| GBT164 | SPBio_000310 | 222.36 |
| GBT165 | Melafolone | 400.46 |
| GBT166 | Methyl 2-chloroacrylate | 120.54 |
| GBT167 | Chlorogenic acid methyl ester | 368.37 |
| GBT168 | (+)-Eudesmin | 386.48 |
| GBT169 | cis-pinosylvin | 212.26 |
| GBT170 | (4S,5S)-4-hydroxy-5-pentyloxolan-2-one | 172.25 |
| GBT171 | Ulmoside | 510.55 |
| GBT172 | Ulmoside_qt | 186.23 |
| GBT173 | (2S)-2-ammonio-6-ureidohexanoate | 189.25 |
| GBT174 | 4-[(2S,3R)-5-[(E)-3-hydroxyprop-1-enyl]-7-methoxy-3-methylol-2,3-dihydrobenzofuran-2-yl]-2-methoxy-phenol | 358.42 |
| GBT175 | hirsutin | 669.67 |
| GBT176 | hirsutin_qt | 345.35 |
| GBT177 | liriodendrin | 742.7 |
| GBT178 | liriodendrin_qt | 450.48 |
| GBT179 | quercetin-3-sophoroside | 626.57 |
| GBT180 | trans-4-Hydroxycyclohexane-1-carboxylic acid | 144.19 |
| GBT181 | quercetin | 302.25 |
| GBT182 | Arachic acid | 312.6 |
| GBT183 | palmitic acid | 256.48 |
| GBT184 | FER | 194.2 |
| GBT185 | linolenic acid | 278.48 |
| GBT186 | Nonacosane | 408.89 |
| GBT187 | lignoceric acid | 368.72 |
| GBT188 | 1-hexanol | 102.2 |
| GBT189 | WLN: VH6 | 114.21 |
| GBT190 | stearic acid | 284.54 |
| GBT191 | LINALOOL (D) | 154.28 |
| GBT192 | n-Triacontanol | 438.92 |
| GBT193 | octanol | 130.26 |
| GBT194 | myristic acid | 228.42 |
| GBT195 | 2,6-Dimethoxyquinol | 170.18 |
| GBT196 | (-)-(7R,8S)-Dihydrodehydrodiconiferyl alcohol | 360.44 |
| GBT197 | (E,7R,11S)-3,7,11,15-tetramethylhexadec-2-en-1-ol | 296.6 |
| GBT198 | NON | 172.3 |
| GBT199 | beta-carotene | 536.96 |
| GBT200 | olivil | 376.44 |
| GBT201 | Damascenone | 190.31 |
| GBT202 | 2-Caren-10-al | 150.24 |
| GBT203 | M-COUMARIC ACID | 164.17 |
| GBT204 | MIPK | 86.15 |
| GBT205 | sorbitol | 182.2 |
| GBT206 | (E)-3-[4-[(1R,2R)-2-hydroxy-2-(4-hydroxy-3-methoxy-phenyl)-1-methylol-ethoxy]-3-methoxy-phenyl]acrolein | 374.42 |
| GBT207 | 3-Ethynylphenol | 118.14 |
| GBT208 | Syringetin | 346.31 |
| GBT209 | Cyt | 111.12 |
| GBT210 | 7β,18,20,26-Tetrahydroxy-20(s)-24E-dammaragonene-3-O-α-L-(3'-acetyl)-arabinopyranose-(l-2)-β-D-glucopyranoside | 827.18 |
| GBT211 | 7β,18,20,26-Tetrahydroxy-20(s)-24E-dammaragonene-3-O-α-L-(4'-acetyl)-arabinopyranose-(l-2)-β-D-glucopyranoside_qt | 490.85 |
| GBT212 | methyl 4-[2-formyl-5-(methoxymethyl)pyrrol-1-yl]butanoate | 239.3 |
| GBT213 | 4-(2-formyl-5-carbooxymethyl-pyrrole-1-yl)-3-phenyl-methyl propanoate | 301.37 |
| GBT214 | daucosterol palmitate | 815.41 |
| GBT215 | beta-sitosterol palmitate | 653.25 |
| GBT216 | Δ7,16,25,26-stigmastatrienol | 410.75 |
| GBT217 | Δ7,16,25,26-stigmastatrienol-3-O-glucoside | 572.91 |
| GBT218 | Δ7,22,25-triene-3-ol | 410.75 |
| GBT219 | Δ7,22,25-stigmastatrienol-3-O-nonadecanoate | 691.3 |
| GBT220 | Δ7,22,25-triene-3-β-D-glucoside | 572.91 |
| GBT221 | 7β,18,20,26-Tetrahydroxy-20(s)-24E-dammaragonene-3-O-α-L-(4'-acetyl)-arabinopyranose-(l-2)-β-D-glucopyranoside | 827.18 |
| GBT222 | Δ7,22,25-stigmastatrienol-3-O-β-D-(6'-palmitoyl)-glucopyranoside | 797.34 |
| GBT223 | 7β,18,20,26-Tetrahydroxy-20(s)-24E-dammaragonene-3-O-α-L-arabinopyranose-(l-2)-β-D-(6'-acetyl)-glucopyranoside | 769.14 |
| GBT224 | 7β,20,26-trihydroxy-20(s)-24E-dammaragonene-3-O-α-L-arabinopyranose-(l-2)-β-D-(6'-acetyl)-glucopyranosi | 474.85 |
| GBT225 | 7β,20,26-trihydroxy-20(s)-24E-dammaragonene-3-O-α-L-arabinopyranose-(l-2)-β-D-(6'-acetyl)-glucopyranoside | 811.18 |
| GBT226 | 7β,20,26-trihydroxy-20(s)-24E-dammaragonene-3-O-α-L-(3'-acetyl)-arabinopyranose-(l-2)-β-D-glucopyranoside | 811.18 |
| GBT227 | 7β,20,26-trihydroxy-20(s)-24E-dammaragonene-3-O-α-L-(4'-acetyl)-arabinopyranose-(l-2)-β-D-glucopyranoside | 825.16 |
| GBT228 | 7β,20,26-trihydroxy-20(s)-24E-dammaragonene-3-O-α-L-(4'-acetyl)-arabinopyranose-(l-2)-β-D-glucopyranosi | 488.83 |
| GBT229 | 7β,20,26-trihydroxy-8-formyl-20(s)-24E-dammaragonene-3-O-α-L-(3'-acetyl)-arabinopyranose-(l-2)-β-D-glucopyranoside | 825.16 |
| GBT230 | 7β,20,26-trihydroxy-8-formyl-20(s)-24E-dammaragonene-3-O-α-L-(4'-acetyl)-arabinopyranose-(l-2)-β-D-glucopyranoside | 1558.07 |
| GBT231 | tubeimoside IV | 787.11 |
| GBT232 | tubeimosideV | 1349.64 |
| GBT233 | 2-ACETYLPYRROLE | 109.14 |
| GBT234 | ADO | 267.28 |
| GBT235 | n-butyl-β-D-fructoufranoside | 236.3 |
| GBT236 | (R)-Allantoin | 158.14 |
| GBT237 | cucurbitacin b | 558.78 |
| GBT238 | Cucurbitacin E | 556.76 |
| GBT239 | Hentriacontan | 436.95 |
| GBT240 | sitosterol | 414.79 |
| GBT241 | emodin | 270.25 |
| GBT242 | 37417-41-7 | 342.34 |
| GBT243 | GLO | 180.18 |
| GBT244 | TRIACONTANE | 422.92 |
| GBT245 | sucrose | 342.34 |
| GBT246 | syringaresinol | 418.48 |
| GBT247 | (+)-Suyringaresinol-di-O-beta-D-glucoside | 742.8 |
| GBT248 | (14S)-14-methylpalmitic acid | 270.51 |
| GBT249 | Acutumidine | 383.86 |
| GBT250 | Acutumine | 397.89 |
| GBT251 | Dispegatrine | 663.96 |
| GBT252 | 16-epi-Isositsirikine | 354.49 |
| GBT253 | Magnograndiolide | 266.37 |
| GBT254 | Michelenolide | 264.35 |
| GBT255 | [[2-(4-hydroxyphenyl)-1-[(2S,3R,4S,5S,6R)-3,4,5-trihydroxy-6-(hydroxymethyl)oxan-2-yl]sulfanylethylidene]amino] hydrogen sulfate | 424.47 |
| GBT256 | Sinomenine | 327.41 |
| GBT257 | Sinomontanine D | 441.58 |
| GBT258 | Stepholidine | 327.41 |
| GBT259 | Tufulingoside | 340.31 |
| GBT260 | ZINC01609418 | 222.41 |
| GBT261 | Hemo-sol | 136.26 |
| GBT262 | alexandrin | 576.95 |
| GBT263 | hederagenin | 414.79 |
| GBT264 | (3S,8S,9S,10R,13R,14S,17R)-10,13-dimethyl-17-[(2R,5S)-5-propan-2-yloctan-2-yl]-2,3,4,7,8,9,11,12,14,15,16,17-dodecahydro-1H-cyclopenta[a]phenanthren-3-ol | 428.82 |
| GBT265 | succinic acid | 118.1 |
| GBT266 | daidzein | 254.25 |
| GBT267 | Ononin | 430.44 |
| GBT268 | formononetin | 268.28 |
| GBT269 | Calycosin | 284.28 |
| GBT270 | (Z)-1-(2,4-dihydroxyphenyl)-3-(4-hydroxyphenyl)prop-2-en-1-one | 256.27 |
| GBT271 | CHEBI:39932 | 128.24 |
| GBT272 | (Z)-1-(2,4-dihydroxyphenyl)-3-(3,4-dihydroxyphenyl)prop-2-en-1-one | 272.27 |
| GBT273 | 3,7-dihydroxy-6-methoxy-dihydroflavonol | 302.3 |
| GBT274 | 3,7-dihydroxy-6-methoxy-dihydroflavonol | 692.98 |
| GBT275 | 16844-71-6 | 428.82 |
| GBT276 | 4-Pipecoline | 99.2 |
| GBT277 | Hydroxysitosterol | 430.79 |
| GBT278 | 6,8-di-C-α-L-arabinosylapigenin | 534.51 |
| GBT279 | Castanin | 298.31 |
| GBT280 | 8-o-Methylreyusi | 298.31 |
| GBT281 | 3-Hydroxystigmast-5-en-7-one | 428.77 |
| GBT282 | 8-C-α-L-arabinosylluteolin | 418.38 |
| GBT283 | aloe-emodin | 270.25 |
| GBT284 | (-)-trans-Carveol | 152.26 |
| GBT285 | (-)-Epoxycaryophyllene | 220.39 |
| GBT286 | anethole | 148.22 |
| GBT287 | Physcion | 284.28 |
| GBT288 | Ethyllaurate | 228.42 |
| GBT289 | Eucarvone | 150.24 |
| GBT290 | Farnesene | 204.39 |
| GBT291 | genistin | 432.41 |
| GBT292 | levodopa | 197.21 |
| GBT293 | (Z)-3-(4-hydroxy-3-methoxy-phenyl)-N-[2-(4-hydroxyphenyl)ethyl]acrylamide | 313.38 |
| GBT294 | Dilospan S | 126.12 |
| GBT295 | TMH | 136.26 |
| GBT296 | Prunetin | 284.28 |
| GBT297 | Schisandrin | 388.5 |
| GBT298 | Schizandrol | 416.51 |
| GBT299 | (1S,4aR,8aR)-1-isopropyl-7-methyl-4-methylene-2,3,4a,5,6,8a-hexahydro-1H-naphthalene | 204.39 |
| GBT300 | petunidin | 317.29 |
| GBT301 | Augelicin | 426.5 |
| GBT302 | campesterol | 400.76 |
| GBT303 | 5-[(3R)-7-hydroxychroman-3-yl]-2,3-dimethoxy-p-benzoquinone | 316.33 |
| GBT304 | odoratin | 344.39 |
| GBT305 | delphinidin-3-glucoside | 465.42 |
| GBT306 | licochalcone a | 338.43 |
| GBT307 | isoorientin | 448.41 |
| GBT308 | malvidin | 331.32 |
| GBT309 | Vestitol | 272.32 |
| GBT310 | Consume close grain | 302.3 |
| GBT311 | Cajinin | 300.28 |
| GBT312 | Medicagol | 296.24 |
| GBT313 | kadsurin | 456.58 |
| GBT314 | interiorin | 486.61 |
| GBT315 | Lupinidine | 234.43 |
| GBT316 | Psi-Baptigenin | 282.26 |
| GBT317 | Friedelin | 426.8 |
| GBT318 | isosativan | 286.35 |
| GBT319 | olmelin | 284.28 |
| GBT320 | Aldrich | 154.18 |
| GBT321 | (L)-alpha-Terpineol | 154.28 |
| GBT322 | dec-2-enal | 154.28 |
| GBT323 | CAM | 152.26 |
| GBT324 | 24-epicampesterol | 400.76 |
| GBT325 | germacrene | 208.43 |
| GBT326 | copaene | 204.39 |
| GBT327 | Linoleyl acetate | 308.56 |
| GBT328 | 24190-29-2 | 192.33 |
| GBT329 | poriferast-5-en-3beta-ol | 414.79 |
| GBT330 | isoliquiritigenin | 256.27 |
| GBT331 | DFV | 256.27 |
| GBT332 | Pulegone | 152.26 |
| GBT333 | (R)-linalool | 154.28 |
| GBT334 | Izosafrol | 162.2 |
| GBT335 | (1S,4R)-fenchone | 152.26 |
| GBT336 | (6R)-6-isopropyl-3-methyl-1-cyclohex-2-enone | 152.26 |
| GBT337 | Methyleugenol | 178.25 |
| GBT338 | tricin | 330.31 |
| GBT339 | alpha-Cubebene | 204.39 |
| GBT340 | 20-Hexadecanoylingenol | 586.94 |
| GBT341 | Terragon | 148.22 |
| GBT342 | ()-Borneol | 154.28 |
| GBT343 | Ginkgetin | 566.54 |
| GBT344 | Isoginkgetin | 566.54 |
| GBT345 | oleanolic acid | 456.78 |
| GBT346 | 3,4,5-Trimethoxytoluene | 182.24 |
| GBT347 | junipene | 204.39 |
| GBT348 | magnoflorine | 342.45 |
| GBT349 | salidroside | 300.34 |
| GBT350 | Tyrosol | 138.18 |
| GBT351 | Ginnol | 424.89 |
| GBT352 | Chryseriol | 300.28 |
| GBT353 | lauric acid | 200.36 |
| GBT354 | Flavone der. | 298.31 |
| GBT355 | (2R,3R)-2-(3,4-dimethoxyphenyl)-7-methoxy-3-methyl-5-[(E)-prop-1-enyl]-2,3-dihydrobenzofuran | 340.45 |
| GBT356 | 8-Isopentenyl-kaempferol | 354.38 |
| GBT357 | Azaron | 208.28 |
| GBT358 | Docosanoate | 340.66 |
| GBT359 | (Z)-heptadec-3-ene | 238.51 |
| GBT360 | 3,5-Dimethoxytoluene | 152.21 |
| GBT361 | Isomenthol | 156.3 |
| GBT362 | Octyl formate | 158.27 |
| GBT363 | Hyperin | 464.41 |
| GBT364 | quercetin-3-rhamnooside | 448.41 |
| GBT365 | Robinetin | 302.25 |
| GBT366 | rouhuoside | 824.86 |
| GBT367 | Sagittatoside A | 676.73 |
| GBT368 | Anhydroicaritin | 368.41 |
| GBT369 | sagittatoside B | 646.7 |
| GBT370 | Trifolin | 448.41 |
| GBT371 | wanepimedoside A | 678.75 |
| GBT372 | wanepimedoside_qt | 386.43 |
| GBT373 | Wushanicariin | 530.57 |
| GBT374 | wushanicariin_qt | 368.41 |
| GBT375 | C-Homoerythrinan, 1,6-didehydro-3,15,16-trimethoxy-, (3.beta.)- | 329.48 |
| GBT376 | Besigomsin | 416.51 |
| GBT377 | Yinyanghuo A | 420.49 |
| GBT378 | Yinyanghuo B | 422.51 |
| GBT379 | Yinyanghuo C | 336.36 |
| GBT380 | Yinyanghuo D | 338.38 |
| GBT381 | Yinyanghuo E | 352.36 |
| GBT382 | Yixinoside A | 1107.49 |
| GBT383 | 6-hydroxy-11,12-dimethoxy-2,2-dimethyl-1,8-dioxo-2,3,4,8-tetrahydro-1H-isochromeno[3,4-h]isoquinolin-2-ium | 370.41 |
| GBT384 | 3-Hexenyl-beta-glucopyranoside | 262.34 |
| GBT385 | 5,7,4'-trihydroxy8,3'-diprenylflavone | 406.51 |
| GBT386 | 8-(3-methylbut-2-enyl)-2-phenyl-chromone | 290.38 |
| GBT387 | acuminatoside | 985.05 |
| GBT388 | anhydroicaritin | 368.41 |
| GBT389 | Anhydroicaritin-3-O-alpha-L-rhamnoside | 676.73 |
| GBT390 | artonin U | 352.41 |
| GBT391 | 1,2-bis(4-hydroxy-3-methoxyphenyl)propan-1,3-diol | 320.37 |
| GBT392 | baohuoside Ⅵ | 822.89 |
| GBT393 | 3,5,7-Trihydroxy-4'-methoxyl-8-prenylflavone-3-O-rhamnopyranoside | 514.57 |
| GBT394 | Baohuoside VI | 822.89 |
| GBT395 | Bilobanol | 234.37 |
| GBT396 | bilobetin | 552.51 |
| GBT397 | brevicornin | 400.46 |
| GBT398 | caohuoside B | 965.01 |
| GBT399 | caohuoside D | 562.62 |
| GBT400 | 3-[(2S,3R,4R,5R,6S)-4,5-dihydroxy-6-methyl-3-[(2S,3R,4S,5S,6R)-3,4,5-trihydroxy-6-methyl-tetrahydropyran-2-yl]oxy-tetrahydropyran-2-yl]oxy-5-hydroxy-2-(4-methoxyphenyl)-8-(3-methylbut-2-enyl)-7-[(2S,3R,4S,5S,6R)-3,4,5-trihydroxy-6-methylol-tetrahydropyran | 822.89 |
| GBT401 | 2,15-Hexadecanedione | 254.46 |
| GBT402 | Epimedin B | 792.86 |
| GBT403 | Epimedin C_qt | 352.41 |
| GBT404 | Epimedin C | 790.89 |
| GBT405 | epimedokoreanone A | 142.12 |
| GBT406 | (2S,3S)-3,5-dihydroxy-2-(4-hydroxyphenyl)-8-(3-methylbut-2-enyl)-7-[(2S,3R,4S,5S,6R)-3,4,5-trihydroxy-6-methylol-tetrahydropyran-2-yl]oxy-chroman-4-one | 518.56 |
| GBT407 | epimedoside C | 516.54 |
| GBT408 | epimedoside D | 794.83 |
| GBT409 | Epimedoside E | 794.83 |
| GBT410 | epimedoside | 760.81 |
| GBT411 | DOB | 154.13 |
| GBT412 | globulol | 222.41 |
| GBT413 | Hentriacontanol-6 | 452.95 |
| GBT414 | hexandraside D | 822.89 |
| GBT415 | Hexandraside E | 678.7 |
| GBT416 | hexandraside F | 838.89 |
| GBT417 | Icaride A2 | 436.5 |
| GBT418 | Icariin | 676.73 |
| GBT419 | Icariresinol | 433.47 |
| GBT420 | Icariside A7 | 462.49 |
| GBT421 | 3,4,6-trimethoxyphenanthrene-2,7-diol | 300.33 |
| GBT422 | icariside C1 | 418.59 |
| GBT423 | icariside I | 530.57 |
| GBT424 | icariside II | 514.57 |
| GBT425 | 4H-1-Benzopyran-4-one, 3-((6-deoxy-alpha-L-mennopyranosyl)oxy)-5,7-dihydroxy-2-(4-hydroxyphenyl)-8-(3-methyl-2-butenyl)- | 500.54 |
| GBT426 | 3-[(2S,3R,4S,5S,6R)-4,5-dihydroxy-6-methylol-3-[(2S,3R,4R,5R,6S)-3,4,5-trihydroxy-6-methyl-tetrahydropyran-2-yl]oxy-tetrahydropyran-2-yl]oxy-5,7-dihydroxy-2-(4-hydroxyphenyl)-8-(3-methylbut-2-enyl)chromone | 662.7 |
| GBT427 | Ikarisoside C | 822.89 |
| GBT428 | Ikarisoside F | 632.67 |
| GBT429 | Ikshusterol | 430.79 |
| GBT430 | Lespedin | 578.57 |
| GBT431 | korepimedoside A | 748.8 |
| GBT432 | korepimedoside B | 965.01 |
| GBT433 | patchouli alcohol | 222.41 |
| GBT434 | apigenin | 270.25 |
| GBT435 | (2r,3r)-4',7-dihydroxy-2',5-dimethoxydihy-droflavonol | 332.3 |
| GBT436 | (2r,3r)-aromadendrin | 288.27 |
| GBT437 | 1,3,7-trihydroxy-2-(3-methylbut-2-enyl)xanthone | 312.3 |
| GBT438 | 3'-o-methylorobol | 300.28 |
| GBT439 | 3-hydroxyblancoxanthone | 394.4 |
| GBT440 | 4',7-dihydroxy-2',5-dimethoxyflavonol | 330.29 |
| GBT441 | 6-deoxyjacareubin | 310.3 |
| GBT442 | alpinumisoflavone | 336.3 |
| GBT443 | alvaxanthone | 396.4 |
| GBT444 | aspidinol | 224.28 |
| GBT445 | bergapten | 216.2 |
| GBT446 | cochinchinol a | 654.8 |
| GBT447 | cochinchinol b | 642.5 |
| GBT448 | cudraflavanone b | 356.4 |
| GBT449 | cudranone | 328.4 |
| GBT450 | cudraphenone a | 364.4 |
| GBT451 | cudraphenone b | 366.4 |
| GBT452 | cudraphenone c | 396.4 |
| GBT453 | cudraphenone d | 382.4 |
| GBT454 | cudraxanthone p | 396.4 |
| GBT455 | cudraxanthone q | 378.4 |
| GBT456 | cudraxanthone r | 412.4 |
| GBT457 | cudraxanthone s | 328.3 |
| GBT458 | gerontoxanthone a | 394.4 |
| GBT459 | gerontoxanthone b | 394.4 |
| GBT460 | gerontoxanthone g | 396.4 |
| GBT461 | gerontoxanthone h | 380.4 |
| GBT462 | gerontoxanthone i | 396.4 |
| GBT463 | isoalvaxanthone | 396.4 |
| GBT464 | isobavachin | 324.4 |
| GBT465 | toxyloxanthone c | 328.3 |
| GBT466 | wighteone | 337.3 |
| GBT467 | angelicin | 186.17 |
| GBT468 | backuchiol | 256.4 |
| GBT469 | bakuchalcone | 340.4 |
| GBT470 | bakuchiol | 242.36 |
| GBT471 | bavachalcone | 324.4 |
| GBT472 | bavachin | 324.4 |
| GBT473 | bavachinin | 336.4 |
| GBT474 | bavachromanol | 356.4 |
| GBT475 | bavachromene | 322.4 |
| GBT476 | bavacoumestan a | 352.3 |
| GBT477 | bavacoumestan b | 352.3 |
| GBT478 | corylidin | 368.3 |
| GBT479 | corylifolinin | 324.4 |
| GBT480 | (+)-medioresinol | 388.45 |
| GBT481 | corylin | 338.4 |
| GBT482 | corylinal | 282.25 |
| GBT483 | daucosterol | 576.95 |
| GBT484 | docosanoicacid | 609.1 |
| GBT485 | isocorylifonol | 234.24 |
| GBT486 | isoneobavachalcone | 324.4 |
| GBT487 | isopsoralidin | 336.3 |
| GBT488 | isposoralen | 186.16 |
| GBT489 | myristicacid | 737.9 |
| GBT490 | neobavaisoflavone | 322.4 |
| GBT492 | neobavachalcone | 298.31 |
| GBT493 | psoraldehyde | 96.13 |
| GBT494 | psoralen | 186.17 |
| GBT495 | psoralenol | 338.4 |
| GBT496 | psoralidin | 336.3 |
| GBT498 | sophoracoumestan a | 334.3 |
| GBT499 | stearicacid | 284.5 |
| GBT500 | (2R,3S)-(+)-3',5-Dihydroxy-4 ,7-dimethoxydihydroflavonol | 332.33 |
