# Supplementary material for: Integrating Network Pharmacology and Experimental Validation to Explore the Key Mechanism of Gubitong Recipe in the Treatment of Osteoarthritis
Source: Comput Math Methods Med. 2022 Jun 8;2022:7858925. doi: 10.1155/2022/7858925 (PMC9200584; doi:10.1155/2022/7858925)
Supplement: Supplementary Materials — Supplementary Table 1: the number of compounds contained in each botanical drug. Supplementary Table 2: the detailed information of compounds contained in GBT. Supplementary Table 3: the information of active compounds in GBT. [file 7858925.f1.zip › Supplymentary Table 3 (1).docx]

**Supplementary Table 3** The information of active compounds in GBT

| **ID** | **MF** | **Name** | **ID** | **MF Name** | |  |
| --- | --- | --- | --- | --- | --- | --- |
| GBT5 | C_15_H_10_O_6_ | Aureusidin | GBT252 | C_21_H_26_N_2_O_3_ | 16-epi-Isositsirikine | |
| GBT12 | C_15_H_12_O_6_ | Eriodyctiol (flavanone) | GBT253 | C_15_H_22_O_4_ | Magnograndiolide | |
| GBT13 | C_29_H_48_O | Stigmasterol | GBT254 | C_15_H_20_O_4_ | Michelenolide | |
| GBT17 | C_29_H_50_O | beta-sitosterol | GBT256 | C_19_H_23_NO_4_ | Sinomenine | |
| GBT19 | C_15_H_10_O_6_ | kaempferol | GBT258 | C_19_H_21_NO_4_ | Stepholidine | |
| GBT20 | C_15_H_12_O_5_ | naringenin | GBT263 | C_30_H_48_O_4_ | hederagenin | |
| GBT24 | C_15_H_14_O_6_ | (+)-catechin | GBT264 | C_30_H_52_O | (3S,8S,9S,10R,13R,14S,17R)-10,13-dimethyl-17-[(2R,5S)-5-propan-2-yloctan-2-yl]-2,3,4,7,8,9,11,12,14,15,16,17-dodecahydro-1H-cyclopenta[a]phenanthren-3-ol | |
| GBT25 | C_15_H_12_O_6_ | eriodictyol | GBT268 | C_16_H_12_O_4_ | formononetin | |
| GBT27 | C_14_H_10_O_9_ | digallate | GBT269 | C_16_H_12_O_5_ | Calycosin | |
| GBT28 | C_15_H_10_O_6_ | luteolin | GBT273 | C_16_H_14_O_6_ | 3,7-dihydroxy-6-methoxy-dihydroflavonol | |
| GBT41 | C_29_H_48_O | 22-Stigmasten-3-one | GBT280 | C_17_H_14_O_5_ | 8-o-Methylreyusi | |
| GBT56 | C_31_H_52_O | cyclolaudenol | GBT281 | C_29_H_48_O_2_ | 3-Hydroxystigmast-5-en-7-one | |
| GBT58 | C_19_H_19_NO_7_ | davallioside A_qt | GBT282 | C_20_H_18_O_10_ | 8-C-α-L-arabinosylluteolin | |
| GBT71 | C_21_H_22_O_5_ | xanthogalenol | GBT283 | C_15_H_10_O_5_ | aloe-emodin | |
| GBT114 | C_20_H_22_O_7_ | AIDS214634 | GBT293 | C_18_H_19_NO_4_ | (Z)-3-(4-hydroxy-3-methoxy-phenyl)-N-[2-(4-hydroxyphenyl)ethyl]acrylamide | |
| GBT132 | C_21_H_24_O_7_ | (+)-medioresinol | GBT300 | C_16_H_13_ClO_7_ | petunidin | |
| GBT163 | C_18_H_20_O_6_ | Helenalin | GBT302 | C_28_H_48_O | campesterol | |
| GBT153 | C_20_H_24_N_2_O_2_ | Cinchonan-9-al, 6'-methoxy-, (9R)- | GBT306 | C_21_H_22_O_4_ | licochalcone A | |
| GBT137 | C_21_H_26_N_2_O_3_ | (-)-Tabernemontanine | GBT309 | C_16_H_16_O_4_ | Vestitol | |
| GBT100 | C_15_H_10_O_6_ | olivil | GBT310 | C_16_H_14_O_6_ | Consume close grain | |
| GBT118 | C_24_H_30_O_8_ | Yangambin | GBT311 | C_16_H_12_O_6_ | Cajinin | |
| GBT85 | C_21_H_24_O_7_ | 40957-99-1 | GBT312 | C_16_H_8_O_6_ | Medicagol | |
| GBT149 | C_27_H_41_NO_2_ | Cyclopamine | GBT315 | C_15_H_26_N_2_ | Lupinidine | |
| GBT206 | C_20_H_22_O_7_ | (E)-3-[4-[(1R,2R)-2-hydroxy-2-(4-hydroxy-3-methoxy-phenyl)-1-methylol-ethoxy]-3-methoxy-phenyl]acrolein | GBT316 | C_16_H_10_O_5_ | Psi-Baptigenin | |
| GBT86 | C_30_H_48_O_3_ | Mairin | GBT383 | C_20_H_20_NO_6_+ | 6-hydroxy-11,12-dimethoxy-2,2-dimethyl-1,8-dioxo-2,3,4,8-tetrahydro-1H-isochromeno[3,4-h]isoquinolin-2-ium | |
| GBT178 | C_28_H_36_O_14_ | liriodendrin_qt | GBT377 | C_25_H_24_O_6_ | Yinyanghuo A | |
| GBT151 | C_19_H_34_O_6_ | Dehydrodiconiferyl alcohol 4,gamma'-di-O-beta-D-glucopyanoside_qt | GBT381 | C_20_H_16_O_6_ | Yinyanghuo E | |
| GBT174 | C_20_H_22_O_6_ | 4-[(2S,3R)-5-[(E)-3-hydroxyprop-1-enyl]-7-methoxy-3-methylol-2,3-dihydrobenzofuran-2-yl]-2-methoxy-phenol | GBT391 | C_17_H_20_O_6_ | 1,2-bis(4-hydroxy-3-methoxyphenyl)propan-1,3-diol | |
| GBT102 | C_18_H_19_NO_3_ | Erythraline | GBT386 | C_20_H_18_O_2_ | 8-(3-methylbut-2-enyl)-2-phenyl-chromone | |
| GBT176 | C_18_H_32_O_7_ | hirsutin_qt | GBT368 | C_21_H_20_O_6_ | Anhydroicaritin | |
| GBT116 | C_15_H_14_O_6_ | ent-Epicatechin | GBT379 | C_20_H_16_O_5_ | Yinyanghuo C | |
| GBT181 | C_15_H_10_O_7_ | quercetin | GBT327 | C_20_H_36_O_2_ | Linoleyl acetate | |
| GBT199 | C_40_H_56_ | β-carotene | GBT389 | C_32_H_52_O_15_ | Anhydroicaritin-3-O-alpha-L-rhamnoside | |
| GBT208 | C_17_H_14_O_8_ | Syringetin | GBT418 | C_33_H_40_O_15_ | Icariin | |
| GBT115 | C_18_H_14_O_4_ | 3-beta-Hydroxymethyllenetanshiquinone | GBT356 | C_20_H_18_O_6_ | 8-Isopentenyl-kaempferol | |
| GBT168 | C_22_H_26_O_6_ | (+)-Eudesmin | GBT375 | C_19_H_39_NO_3_ | C-Homoerythrinan,1,6-didehydro-3,15,16-trimethoxy-, (3.beta.)- | |
| GBT130 | C_27_H_34_O_12_ | Eucommin A | GBT324 | C_28_H_48_O | 24-epicampesterol | |
| GBT438 | C_16_H_12_O_6_ | 3'-o-methylorobol | GBT329 | C_29_H_50_O | poriferast-5-en-3beta-ol | |
| GBT464 | C_20_H_20_O_4_ | isobavachin | GBT331 | C_15_H_12_O_4_ | DFV | |
| GBT486 | C_17_H_14_O_5_ | isoneobavachalcone | GBT352 | C_16_H_12_O_6_ | Chryseriol | |
| GBT215 | C_45_H_80_O_2_ | beta-sitosterol palmitate | GBT420 | C_23_H_26_O_10_ | Icariside A7 | |
| GBT216 | C_29_H_46_O | Δ7,16,25,26-stigmastatrienol | GBT480 | C_21_H_24_O_7_ | (+)-medioresinol | |
| GBT218 | C_29_H_46_O | Δ7,22,25-triene-3-ol | GBT489 | C_17_H_14_O_5_ | neobavachalcone | |
| GBT240 | C_29_H_50_O | sitosterol |  |  | |  |
